# The Role of Ethanol in Lithium-Mediated Nitrogen Reduction

**DOI:** 10.1021/jacs.5c03389

**Published:** 2025-08-11

**Authors:** Olivia Westhead, Romain Tort, James O. Douglas, Michele Conroy, Bethan J. V. Davies, Anna Winiwarter, Aishah Faisal, Matthew Spry, Artem Khobnya, Mary P. Ryan, Maria-Magdalena Titirici, Rhodri Jervis, Ifan E. L. Stephens

**Affiliations:** † Department of Materials, 4615 Imperial College London, South Kensington, London SW7 2AZ, U.K.; ‡ Department of Chemical Engineering, 4615 Imperial College London, South Kensington, London SW7 2AZ, U.K.; § Electrochemical Innovation Lab, Department of Chemical Engineering, 4919 University College London, Torrington Place, London WC1E 7JE, U.K.; ∥ Advanced Propulsion Lab, Marshgate, University College London, Stratford E20 2AE, U.K.

## Abstract

Although the Haber–Bosch process for industrial ammonia production is hailed by many as one of the most influential breakthroughs of the 20th century, its decarbonization and decentralization remain a critical challenge. One of the most promising and fast improving approaches is electrochemical nitrogen reduction mediated by lithium. However, the impact of electrolyte configuration on the formation of the solid electrolyte interphase (SEI) and its effect on selective nitrogen reduction is still elusive. In particular, the role of commonly added, supposedly sacrificial, proton donors on SEI chemistry and morphology remains a mystery. In this work, the impact of ethanol concentration in a 1 M LiNTf_2_ in THF electrolyte on SEI properties and nitrogen reduction is analyzed via a multipronged characterization approach. Post-mortem surface analysis via X-ray photoelectron spectroscopy shows a dependence in the relative proportion of LiF and Li_2_O on ethanol concentration, while depth profiling measurements via cluster source time-of-flight secondary ion mass spectrometry reveal increasing SEI electrolyte permeability at higher ethanol concentrations. Cryogenic electron microscopy measurements show a reduction in SEI thickness with increased ethanol concentration, as well as increased SEI homogeneity. Lithium metal is also observed only in the ethanol-free condition. Analysis of bulk SEI components via titration corroborates the observation of lithium metal in cryo-microscopy measurements, as well as showing an increase in bulk Li_2–*x*_OH_*x*_ content with ethanol concentration. A narrow ‘Goldilocks’ region is revealed, where the SEI has just the right properties for efficient nitrogen reduction.

## Graphical abstract

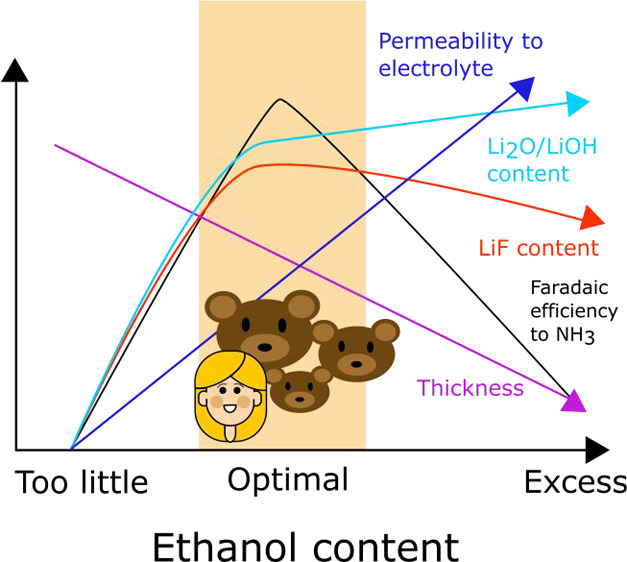

## Introduction

First developed in the early 20th century, the Haber-Bosch process to make ammonia has been instrumental in supporting the growth of approximately half of the world population via nitrogen-based fertilizers.^1^ Ammonia has further potential as a carbon free, readily liquified, and energy dense fuel.^2^ However, while efficient and well-optimized, the Haber-Bosch process is both extremely energy and carbon intensive. Haber-Bosch ammonia plants primarily rely on methane steam reforming to obtain hydrogen gas, resulting in approximately 1.4% of global CO_2_ emissions and the use of 1% of global energy requirements.^1^ Furthermore, the reaction requires extreme operation conditions (400 °C, 200 bar) to improve reaction kinetics,^3^ restricting Haber-Bosch ammonia production to large, centralized plants due to economies of scale. Centralized ammonia production results in fertilizer inequity based on wealth and geography: Countries which lack the capital to build their own Haber-Bosch facility or the infrastructure to transport ammonia tend to suffer more on the global hunger index.^1,4^ A distributed, sustainable mode of ammonia synthesis would be preferable, such as electrochemical ammonia synthesis by nitrogen reduction.^5^

The sole examples of rigorously verified continuous electrochemical nitrogen reduction on a solid electrode are the nonaqueous lithium and calcium mediated nitrogen reduction systems,^6–9^ which both follow the same general principle. In these systems, a lithium or calcium salt is used to generate an active surface in situ via electrodeposition in an organic, aprotic solvent. In analogy to lithium-ion batteries, a Solid Electrolyte Interphase (SEI) layer forms on top of the active surface which consists of the decomposition products of the organic electrolyte.^3,10–12^ This SEI layer serves not only as protection from further electrolyte degradation but also regulates the access of key reactants (nitrogen, protons, and metal ions) to the active surface.^10,13–21^ One key aspect of the lithium-mediated nitrogen reduction electrolyte is the proton donor, which has garnered significant attention recently.^10,18,22–24^ The most common electrolyte in the lithium-mediated nitrogen reduction system is tetrahydrofuran (THF) based, with a small quantity of ethanol added as a proton donor, although there have been investigations into other solvents^25^ and proton donors.^22^ Until recently, the conventional wisdom was that ethanol acted as a sacrificial proton donor.^8,26^ However, Fu and Pedersen et al. revealed with online mass spectrometry that ethanol may also be acting as a proton shuttle, delivering protons produced at the anode (in this case from hydrogen oxidation) to the cathode.^27^ Furthermore, Mygind et al. suggest that ethanol may not be required for ammonia synthesis after SEI formation.^23^ Steinberg et al. revealed markedly different SEI morphology with and without the addition of ethanol to a LiBF_4_ in THF based electrolyte by cryo-transmission electron microscopy (TEM) measurements and suggest that the addition of ethanol activates the SEI for nitrogen reduction by generating organic SEI species which are poorly passivating. This allows nitrogen and protons to access the active surface, with porosity generated by hydrogen evolution.^10^ In operando grazing incidence wide-angle X-ray scattering measurements have also detected the presence of lithium ethoxide in the SEI, suggesting a chemical impact on the SEI from ethanol.^28^ It therefore seems that ethanol plays a more complex role in lithium-mediated nitrogen reduction than originally thought.

A reoccurring theme in the literature is the presence of a volcano-like relationship between electrolyte parameters and nitrogen reduction performance. This was noted in our group upon varying the electrolyte salt concentration^21^ and the trace water concentration,^19^ as well as by others by varying trace oxygen content.^16^ The same is true of ethanol concentration, as has been noted by multiple groups.^8,9,29^ The fact that this shape appears repeatedly in the literature suggests some underlying phenomenon that is controlling behavior, although the complexity of the lithium-mediated nitrogen reduction system makes it difficult to attribute behavior to any one component. On the basis of previous studies published by both our group and others, we hypothesize that the nature of the SEI - rather than exhibiting a binary difference between with and without ethanol - incrementally changes with ethanol concentration. Therefore, it is imperative to establish which characteristics lead to the optimum SEI chemistry and morphology. Herein, we explore these characteristics in a 1 M LiNTf_2_ (LiN­(SO_2_CF_3_)_2_), in THF electrolyte, which earlier studies had identified to lead to relatively high Faradaic efficiency for N_2_ reduction.^30^ We complemented our N_2_ reduction tests, with detailed ex-situ characterization of the SEI, composition and morphology, in particular: post-mortem cryo- electron microscopy, X-ray photoelectron spectroscopy (XPS), time-of-flight secondary ion mass spectrometry (ToF-SIMS), and chemical composition analysis by liquid phase reaction (referred to as SEI titration^31,32^ ) measurements. Through these measurements we correlate key trends in SEI properties with electrochemical performance. We anticipate that such trends can be applied to lithium mediated nitrogen reduction in any electrolyte, cell geometry, or testing condition.

## Results and Discussion

### Electrochemistry

Figure 1 shows the electrochemical results obtained from the reduction of N_2_ on Li plated on a Mo current collector from a 1 M LiNTf_2_ in THF electrolyte with various ethanol concentrations. Figure 1a shows the chronopotentiometry data obtained for the 0 (pink), 34 (purple), and 86 (green) mM ethanol experiments (0, 0.2, and 0.5 vol %, respectively), which from Figure 1b represent too little ethanol (0 mM), a close to optimal ethanol concentration (34 mM) and too much ethanol (86 mM). 10 C (Coulomb) of charge were passed for every experiment, a value chosen as a compromise between passing enough charge to reach steady ammonia Faradaic efficiency,^18,29^ and preventing artifacts from contribution of solvent oxidation products to the performance at the negative electrode at too high amounts of charge passed. While the choice of current collector affects the Faradaic efficiency of ammonia synthesis,^8,30^ and has also been reported to affect the SEI chemistry in anode-less Li metal batteries,^33^ these effects are typically supplanted by the variations in Faradaic efficiency associated with variations in electrolyte composition.^30^ Hence, we did not investigate different current collectors. Again, there is the same volcano-like relationship between the concentration of electrolyte components and nitrogen reduction performance, as has been previously reported in literature.^16,19,29^ The maximum obtained Faradaic efficiency and yield rate toward ammonia were 32 ± 2% and 2.2 ± 0.1 nmol cm^–2^ s^–1^, respectively. This peak is relatively sharp, since an increase of only 69 mM results in a loss of approximately two-thirds of the Faradaic efficiency. This highlights the sensitivity of the lithium-mediated nitrogen reduction system to its electrolyte components.^19,20^

**Figure 1:**
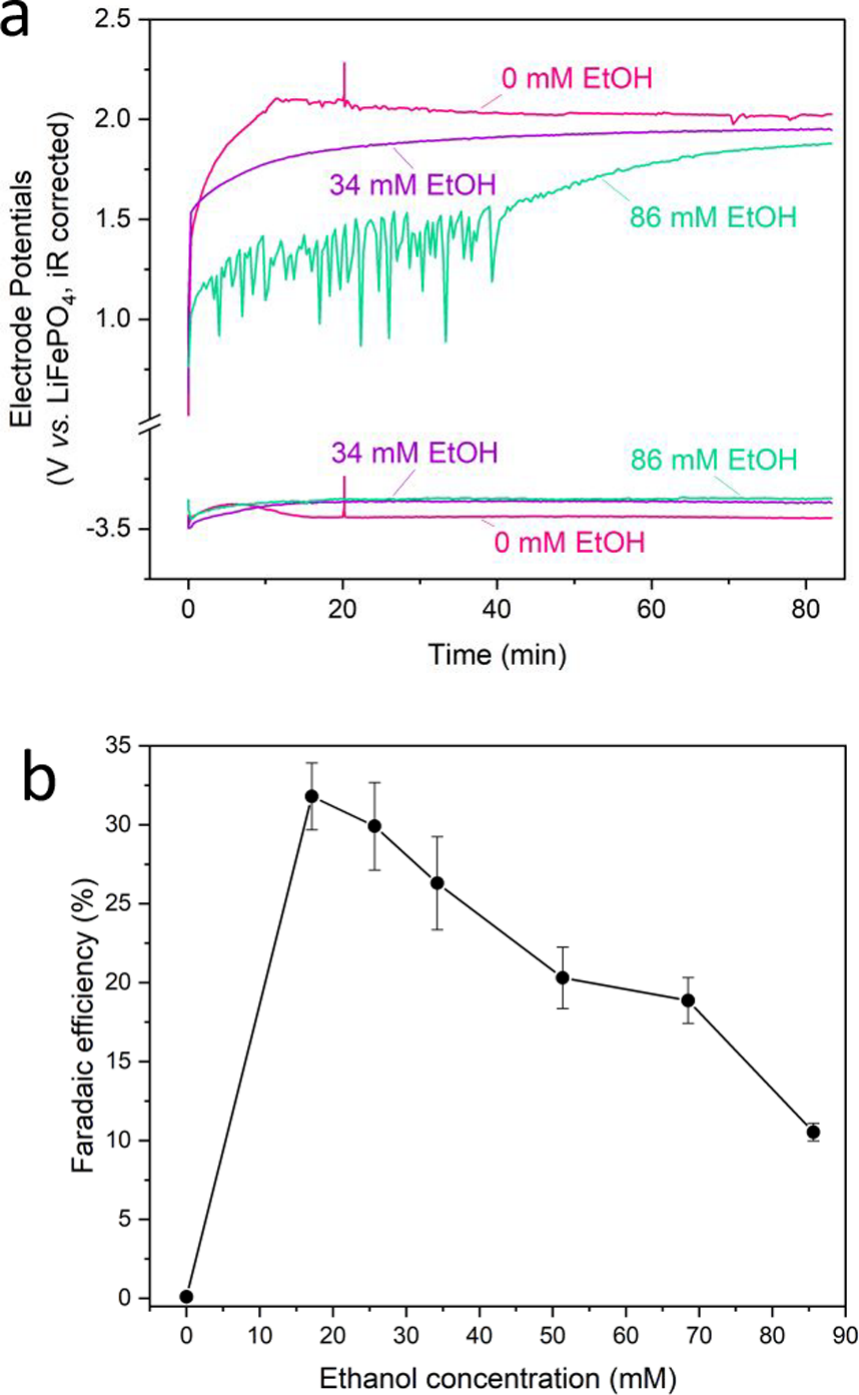
Variation in electrochemical performance of a 1 M LiNTf_2_ in THF electrolyte at 1 bar N_2_ with varying concentrations of ethanol. (a) Variation in working (Mo foil, WE) and counter (Pt mesh, CE) electrode potentials vs a LiFePO_4_ reference, under a constant applied current density of −2 mA cm^–2^ until −10 C is passed. All potentials are corrected for ohmic drop. Pink = 0 mM (0 vol %), purple = 34 mM (0.2 vol %), and green = 86 mM (0.5 vol %) ethanol. (b) Variation in Faradaic efficiency toward ammonia after passing −10 C at a constant applied current density of −2 mA cm^–2^ (*n* = 3). Electrochemistry and quantification methods are shown in Figures S1 and S2.

Figure 1a shows that there is little variation in the working electrode potential between 34 and 86 mM EtOH. Although our previous study on ethanol concentration in a LiClO_4_ containing electrolyte showed significant variation in working electrode potential between 86 and 857 mM (0.5 and 5 vol %) EtOH,^34^ it may be that the change in ethanol concentration presented in Figure 1 was too little to illicit a significant change in operating potential. The 0 mM ethanol condition did however result in a more unstable and negative working electrode potential, which may be due to excessive electrode passivation. Figure S3a,b show the difference in the electrolyte color before and after electrochemistry for the 0 and 86 mM ethanol conditions, respectively. While the 0 mM ethanol electrolyte is significantly discoloured post electrochemistry, the 86 mM ethanol is not, although it is cloudier. This is likely due to uncontrolled electrolyte oxidation at the anode, as has previously been reported.^35^ This observation is consistent with the decrease in counter electrode potential with increasing EtOH concentration (Figure 1a). This decrease in potential could be attributed to a switch from solvent oxidation to ethanol oxidation,^35^ or rather a decrease in Pt poisoning by oligo-/poly-THF species, since ethanol was shown to quench the cationic polymerization of THF, slowing down the rate Pt poisoning with THF degradation products.^24,36,18,25,30^

### Cryo-Microscopy

In order to investigate the impact of ethanol concentration on SEI morphology, SEIs generated in three different ethanol concentrations were analyzed by cryogenic scanning electron microscopy (SEM) and focused ion beam (FIB) milling. These were 0, 26, and 86 mM ethanol to represent the two extremes and a close to optimal ethanol concentration. In later characterizations, this “close to optimal” point was allocated to different absolute amounts of ethanol (either 17, 26, or 34 mM). However, they all represent an intermediate ethanol concentration (between 0 and 86 mM) and should be representative of the trend we aim to elucidate in this work. Cryogenic conditions were required due to the instability of the lithium-mediated nitrogen reduction SEI under the electron beam (Figure S4). All the cross sections presented herein exhibit some degree of curtaining, an artifact due to nonuniform milling rates from compositional or structural variations, causing cross sections to have a pillar like morphology, similar to a hanging curtain. The curtaining arose due to the difficulty of depositing a protection layer under cryogenic conditions and the inhomogeneous and porous nature of the observed SEI samples.^37^ Full experimental details are provided in the Supporting Information. Figure S6 shows the SEM micrographs generated of the SEI surface in the three different ethanol concentrations, with the bare Mo surface shown in Figure S5. The SEI generated in the 0 mM ethanol condition (Figure S6a) differed significantly from the ethanol-containing conditions (Figure S6b,c). In the 0 mM ethanol condition, which was the only condition in which electrode deposits were visible to the naked eye, a heterogeneous morphology was observed. Large islands (on the order of 100 μm in diameter) and some craters are visible, with a rough morphology also visible across the whole SEI area. For the 26 and 86 mM conditions, the electrode deposits were not visible to the naked eye, and the SEI surfaces were smooth and fairly homogeneous. Any observed topographical changes in the SEM micrographs are likely due to dried salt.

Figure 2 shows the SEM micrographs of focused ion beam (FIB) cross sections of the SEI formed in a 0 mM ethanol electrolyte. Figure 2a shows the full SEI cross section, which is extraordinarily thick, on the order of 60 μm. A similar thickness was observed in a separate sample generated in an ethanol free electrolyte (Figure S4a–c). Conversely, lithium-ion battery SEIs have thicknesses on the order of a few nanometers.^12^ There are various areas of differing contrast with the cross section, as well as large voids.

**Figure 2:**
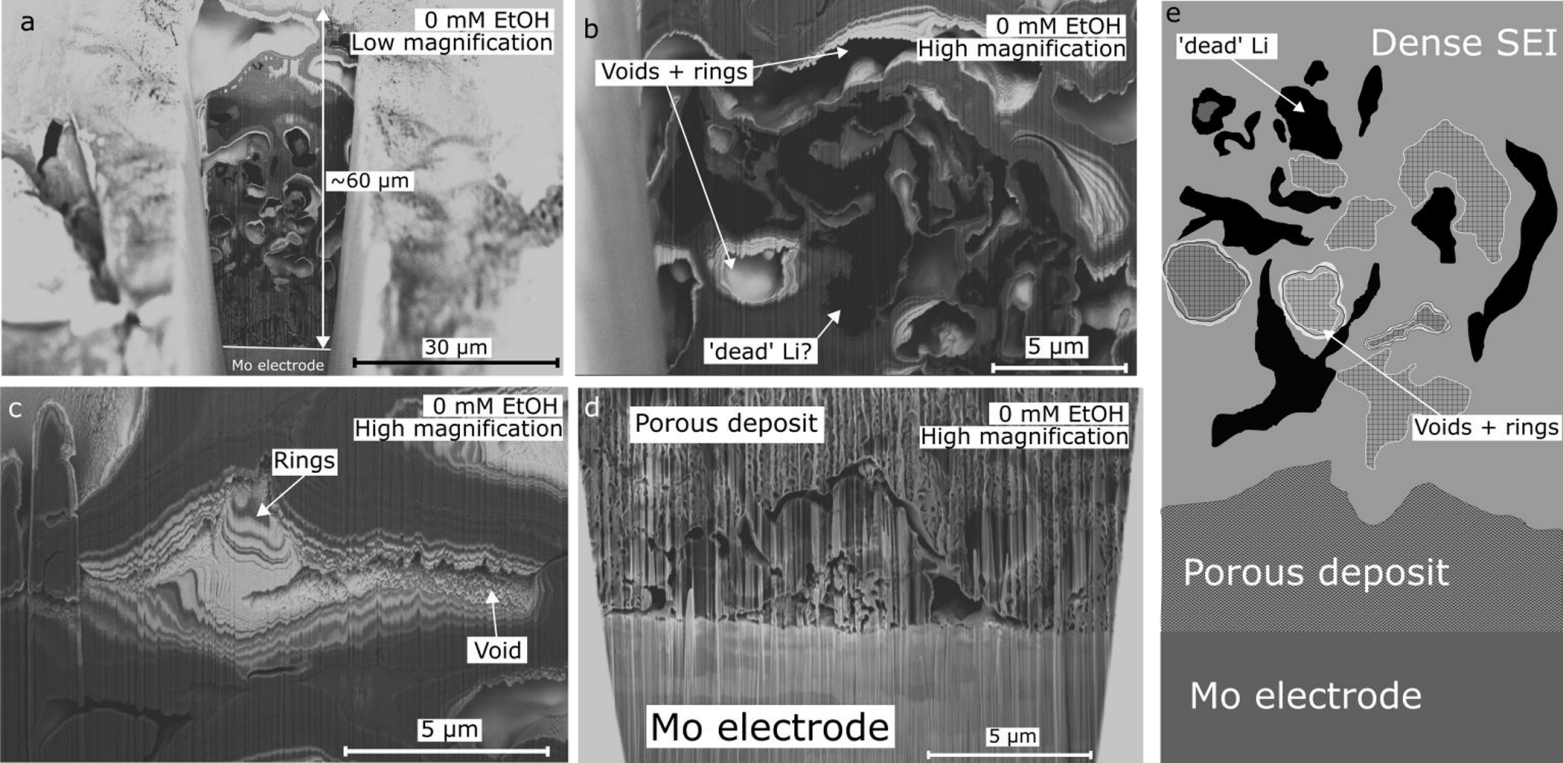
Scanning electron microscopy micrographs of solid electrolyte interphase (SEI) cross sections obtained by focused ion beam milling in the 0 mM ethanol condition (1 M LiNTf_2_, THF as majority solvent) after −10 C was passed at a constant current of −2 mA cm^–2^ on a Mo working electrode. <10 μL THF was drop-cast on the electrode inside an N_2_ glovebox prior to plunge freezing in liquid nitrogen inside the glovebox and then transfer under cryogenic conditions and vacuum to the microscope. Micrographs a-c were taken using the backscatter detector, while micrograph d was taken using the secondary electron detector. (a) Full SEI cross section, where the light contrast at the bottom of the micrograph is the Mo electrode. (b) Zoomed in micrograph of the areas of black contrast and voids in the full SEI cross section. (c) Zoomed in micrograph of a void surrounded by rings of different contrast. (d) Zoomed in micrograph of the area close to the electrode surface. The lighter contrast area with large grains at the bottom of the micrograph is the Mo electrode. (e) Simplified schematic of the 0 mM ethanol SEI, showing the porous inner SEI and the dense outer SEI containing dead Li (solid black) and voids (hatched areas) surrounded by rings of differing contrast.

Figure 2b shows a zoomed in micrograph of the cross section, highlighting the presence of extremely dark and light contrast, suggesting differences in chemical composition, as well as voids. It is possible that the areas of extremely dark contrast may be ‘dead’ lithium, since areas of dark contrast suggest the presence of a low-atomic number element when imaging using backscattered electrons.^38^ Similar areas of dark contrast were also observed in an SEI sample generated in a separate electrochemical experiment in the absence of ethanol (Figure S4c). In a lithium-ion battery, dead lithium is metallic lithium which has become disconnected from the electrode surface and is inactive for energy storage.^38^ In lithium mediated nitrogen reduction, the fate of ‘dead’ lithium is less certain. In theory, even lithium which is disconnected from the electrode surface could still make ammonia chemically via the reactions displayed in eqs 1 and 2:

$$6\mathrm{Li}+N_{2}\to 2{\mathrm{Li}}_{3}N$$ 1

$${\mathrm{Li}}_{3}N+3H^{+}\to {\mathrm{NH}}_{3}+3{\mathrm{Li}}^{+}$$ 2

Therefore, if exposed to nitrogen and protons, any metallic lithium would decompose to form ammonia and lithium ions. If metallic lithium was connected to the electrode surface, the required electrons for the reaction could come from the electrode rather than the lithium itself, allowing it to stay metallic, or it could be quickly rereduced to form metallic lithium again. However, isolated in the SEI, it is unlikely that lithium which has access to nitrogen and protons would remain metallic. Therefore, the fact that possible metallic lithium is observable in the SEI cross sections shown in Figure 2 could suggest a lack of transport of protons and nitrogen through the SEI, as suggested by Steinberg et al.^10^ This would directly result in a negligible Faradaic efficiency toward ammonia. However, without a more chemically sensitive technique it is difficult to know for certain whether these dark areas are indeed metallic lithium.

Figure 2c shows a zoomed in micrograph of a small void which is surrounded by rings of darker and lighter contrast. As shown in Figure 2a,b, the SEI cross section has multiple different voids, which may have formed due to the presence of less stable SEI components which dissolved upon the removal of a reducing potential or may have been filled with electrolyte during the electrochemical measurement. The ring like features surrounding the voids could provide evidence for layered deposition of different SEI components, which would eventually fill the void. Indeed, in Figure 2c, it appears that only a small void may remain. This, along with the fact that the SEI is so thick, suggests continued SEI growth and instability over the course of an experiment. This would likely result in reduced Faradaic efficiency to ammonia.

Figure 2d shows the interface of the Mo electrode (shown in lighter contrast) and a porous network of electrode deposits. This part of the cross section looks quite different to the SEI layer further away from the electrode surface, which is clearly visible in Figure 2a. It is unclear as to exactly what this porous structure may be. It could be mossy lithium deposits on the electrode surface, which would make sense given that this morphology is only visible at the electrode interface. However, this porous network has a lighter contrast than the dark spots visible in Figure 2a,b. Given that elements of lower atomic number appear darker, and lithium is a very light element, it is unclear exactly what material would exhibit a darker contrast in this SEI. Since material density can also play a role in contrast in SEM micrographs, it could be the porous network close to the electrode surface and the darkest spots in the SEI are both lithium, but of differing density. However, without a chemically sensitive technique, it is impossible to know for certain what this layer is.

Figure 2e shows a simplified schematic of the whole SEI thickness, highlighting the key features of the porous, quite uniform, deposit close to the electrode surface and the dense outer SEI containing possible dead lithium, as well as voids and ring like deposits.

Figure 3 shows SEM micrographs of the cross sections of SEIs formed in 26 (a,b) and 86 (d,e) mM ethanol containing electrolytes. These SEI cross sections are much more homogeneous than those formed in the 0 mM electrolyte (Figure 2), with no dark spots suggesting the presence of possible ‘dead’ lithium. The SEIs formed in the ethanol containing electrolytes are much thinner than those formed in the 0 mM ethanol electrolyte, with the 26 mM ethanol SEI having a cross section on the order of 10 μm thick (Figure 3a), and the 86 mM ethanol SEI with thickness on the order of 1–3 μm (3d and e). Both exhibit porosity which appears to be relatively homogeneous with depth. Figure 3a shows the presence of three parallel cracks, which may be an artifact of sample preparation or the cooling process. A very dense network of small pores is also visible, as shown in more detail in Figure 3b. Figure S4 shows results from a separate 26 mM ethanol sample, which has a similar morphology except for the presence of several large voids, the largest of which appears vulnerable to charging as shown by the bright spot inside the void. This could provide weak evidence of a different chemistry inside the void than the rest of the cross section, but a chemically sensitive technique would be required to know for certain. The 86 mM ethanol SEI is thin and porous, as shown in Figure 3d,e. Although Figure 3d is highly curtained, it is possible to see the dense network of small pores which exist within the SEI cross section. Differences in contrast here are due to the morphology of the cross section rather than chemistry. Such a thin and porous SEI is unlikely to be able to sufficiently regulate proton access to the active surface, likely resulting in preferential hydrogen evolution over nitrogen reduction. Figure 3c,f show simplified schematics of the two SEI cases, both showing dense, uniform porosity but differing in thickness.

**Figure 3:**
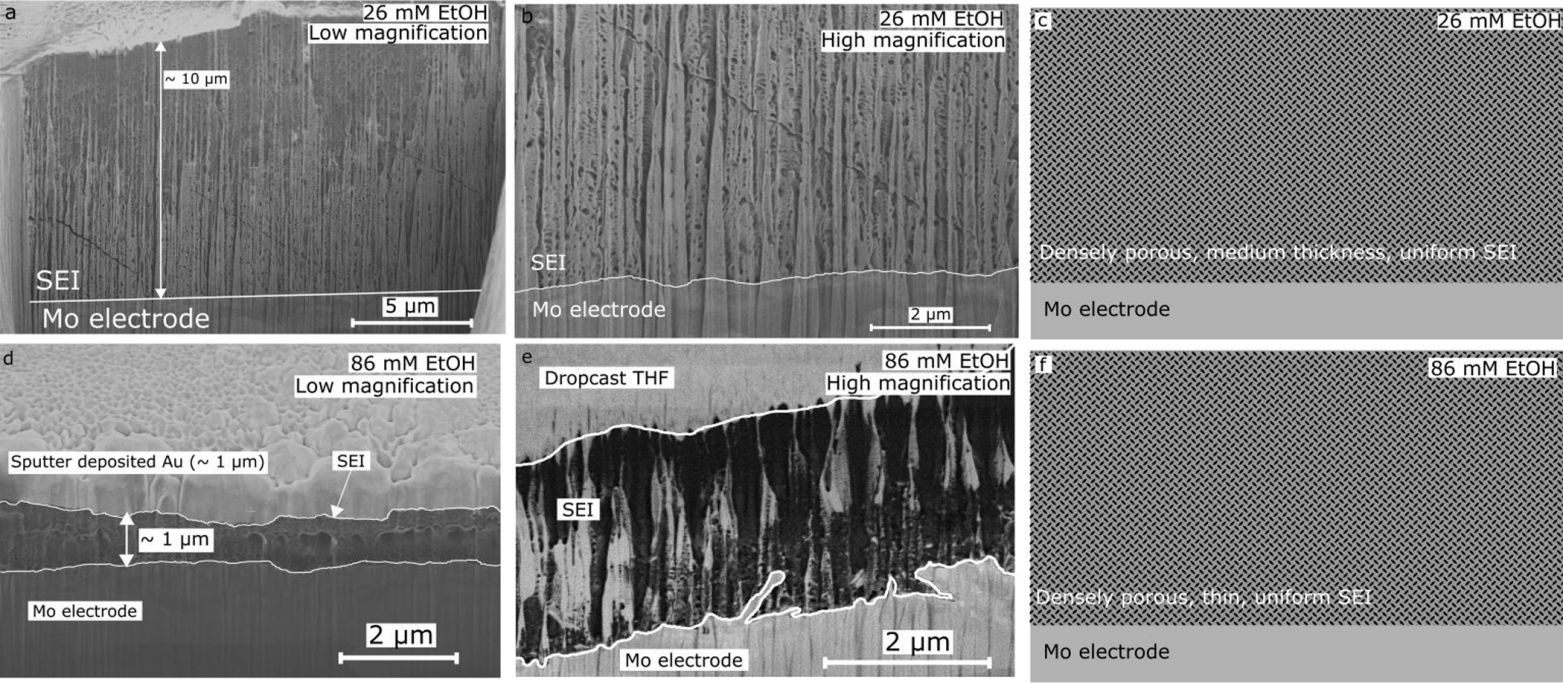
Scanning electron microscopy micrographs of solid electrolyte interphase (SEI) cross sections obtained by focused ion beam milling in (a, b) 26 and (c, d) 86 mM ethanol electrolyte (1 M LiNTf_2_, THF as majority solvent) after −10 C was passed at a constant current of −2 mA cm^–2^ on a Mo working electrode. (a) Cross section of the porous SEI formed in 26 mM ethanol. The Mo is visible at the bottom of the micrograph with large grains. (b) More zoomed in image of the interface between the SEI and Mo electrode on the same cross section as (a). (c) Simplifies the schematic of the 26 mM EtOH SEI, showing uniform dense porosity throughout with no evidence for dead Li. (d) Cross section of the SEI formed in 86 mM ethanol. <10 μL of THF was dropcast onto the sample prior to immersion in liquid nitrogen inside an N_2_ glovebox and transfer to the microscope under vacuum and cryogenic conditions. The THF is visible as the light contrast at the top of the micrograph. The Mo electrode is visible as the lighter contrast at the bottom of the micrograph. (e) Higher magnification cross section of a different SEI sample formed in 86 mM ethanol. This sample was coated with 1 μm Au by sputter deposition without air exposure prior to cryo-microscopy (shown by the very light contrast at the top of the cross section). The Mo is visible at the bottom of the cross section. Micrographs were all taken using the secondary electron detector, except for (d), which was taken with the backscattered electron detector. (f) Simplified schematic of the 86 mM EtOH SEI, showing uniform dense porosity throughout with no evidence for dead Li. The SEI is much thinner than the 26 mM EtOH case. Parallel cracks in (a, b) may be artifacts resulting from the sample preparation or cooling process.

Further cryo-microscopy measurements support the micrographs shown here (see supplementary discussion and Figure S4); areas of dark contrast are only visible in the 0 mM ethanol condition, and the SEI thickness decreases with increasing ethanol content.

In summary, it appears that the concentration of ethanol greatly affects the morphology of the formed SEI. The lack of ethanol results in an enormously thick SEI layer, perhaps with some metallic lithium both at the electrode interface and within the SEI bulk as ‘dead’ lithium. The introduction of ethanol reduces the SEI thickness and increases homogeneity, with no evidence for the presence of metallic lithium in ‘dead’ or ‘active’ form. However, the SEI formed in all three cases had thickness orders of magnitude larger than battery SEIs, which tend to have thickness on the nanometer scale.^12^ Furthermore, while the ethanol containing SEI samples have porosity visible throughout the depth of the SEI, the ethanol free sample appears relatively dense above the porous network visible at the electrode interface. It is likely that porosity and reduced thickness of the ethanol containing SEIs allowed for faster reactant transport to the active surface.

The SEIs observed in this work are also much thicker than those observed by Steinberg et al., who reported roughly 400 nm thick electrode deposits (a thick metallic Li layer covered with a thin 20–30 nm SEI) for an ethanol free SEI and thinner deposits in the presence of ethanol. Steinberg et al. also reported different Faradaic efficiencies to ammonia than those reported in this work.^10^ While it is difficult to compare absolute Faradaic efficiencies across studies, it is worth keeping in mind that differences in performance and in SEI behavior can be expected from working with different systems (e.g., Li salt, cell configuration, current densities, ···). In batteries, the thickness of the SEI is limited by how electronically passivating it is a thinner SEI means an SEI which provides better resistance to continued electrolyte degradation.^12^ It appears that the thickness of the SEIs examined in this work are not limited by electron transport. Therefore, it seems that the interplay between salt choice (in this work LiNTf_2_, and LiBF_4_ for Steinberg et al.^10^) and ethanol concentration is critical to modulating how passivating an SEI is formed, both in terms of limiting continued SEI formation and balancing the transport of nitrogen reduction reactants.

### Surface SEI Chemistry

Figure 4 shows XPS results obtained for the SEI formed in electrolytes containing different ethanol concentrations. All samples were gently rinsed in 0.1 mL THF prior to analysis to remove as much dried salt as possible. However, some dried salt will inevitably remain on the surface (Figure S8d). Figure 4a shows the change in the relative atomic concentrations of F, Li, O, C, S and N from the integrals of their respective core level spectra. Interestingly, the shape of the change in F 1s concentration is similar to that of the Faradaic efficiency (Figure 1b), with the maximum fluorine concentration occurring close to 17 mM ethanol. The measurement of the 26 mM ethanol condition was repeated on a separate sample due to its deviation from the trend. Interestingly, the trend deviation for the 26 mM sample was relatively reproducible, with a dip in the F 1s and an increase in the Li 1s and O 1s atomic concentrations. XPS samples were also measured at ethanol concentrations of 22 and 30 mM in order to further strengthen the observed trend.

**Figure 4:**
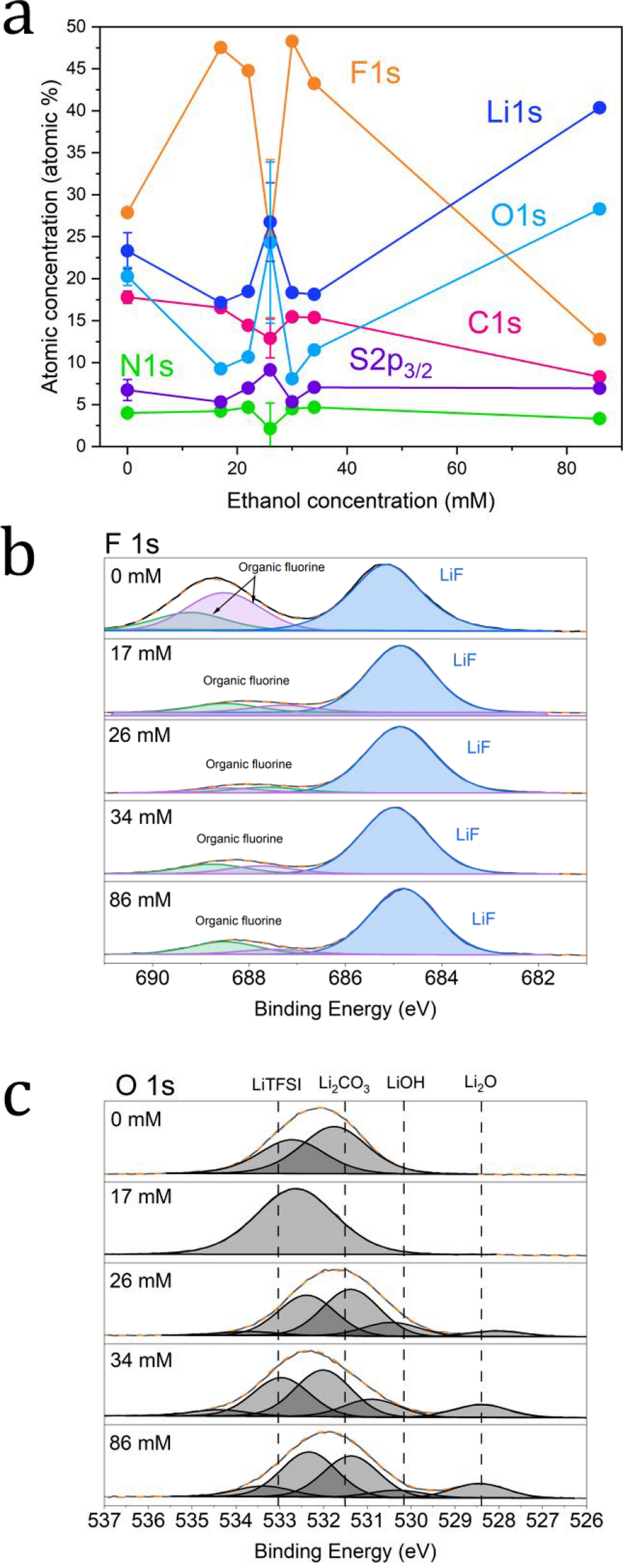
X-ray photoelectron spectroscopy results of the solid electrolyte interphase (SEI) formed on a Mo working electrode after passing −10 C at −2 mA cm^–2^ under 1 bar N_2_ in a 1 M LiNTf_2_ in THF electrolyte with varying ethanol content. All core level spectra are normalized to the maximum for that spectrum. Fitting parameters and survey spectra (Figure S7) are presented in the Supporting Information. Samples were transferred to the spectrometer without air exposure. (a) Variation in the atomic concentration of F, Li, O, C, S, and N from 0 to 86 mM ethanol. The error bars on the 0 mM data points represent the standard deviation in the measurement of two spots on the same sample, while the error bar on the 26 mM data point represents the standard deviation of the measurement of two separate samples. (b, c) F 1s and O 1s core level spectra, respectively. The Li 1s and N 1s core level spectra had no clear features but were fitted to provide the relative atomic concentration (Figure S8).

Figure 4b shows the F 1s core level spectra for the five ethanol concentrations considered. The addition of ethanol results in a much greater ratio of LiF to organic fluorine; at 0 mM ethanol, the ratio of LiF to organic fluorine is 56 ± 3:44 ± 3 (*n* = 2 spots measured). At 17 mM, this ratio increases to 81:19. Except for the 26 mM condition, where the ratio increases to 88 ± 3:12 ± 3 (*n* = 2 samples measured), the relative proportion of LiF to organic fluorine remains constant at approximately 80:20. However, from Figure 4a, the reduction in the relative atomic concentration of C 1s does not exactly match the increase in fluorine content. This finding could suggest that the absolute quantity of organic fluorine is not changing significantly, but the amount of LiF is. This finding is similar to that of Nguyen et al.,^39^ who found that the ratio of −CF_3_ to LiF in the SEI directly correlated to lithium-mediated nitrogen reduction performance in an LiNTf_2_ containing electrolyte.

These data suggest that the quantity of LiF present in the SEI has an impact on the Faradaic efficiency of lithium-mediated nitrogen reduction. Indeed, LiF has been cited as a vital component of the lithium-ion battery SEI and has been linked to the inhibition of hydrogen evolution.^40,41^ Li et al. proposed that in lithium-mediated nitrogen reduction, LiF is proposed to allow for slower and more uniform lithium deposition and improved protection against electrolyte degradation.^17^ Nguyen et al. also suggested that the presence of LiF allows for more favorable lithium deposition morphology.^39^ Furthermore, DFT calculations have suggested that the presence of LiF can reduce the energy barrier toward nitrogen protonation.^42^

Figure 4c shows the O 1s core level spectra for the five ethanol concentrations considered. Due to the small chemical shift in the O 1s core level, it is difficult to confidently assign peaks; it could be that there are multiple species hidden within one fitted peak, which can cause skewing of peak positions. For this reason, we fit the spectra with the minimum possible number of peaks to avoid overfitting the data. Therefore, the 17 mM O 1s spectrum is fitted with a single peak, which may in fact contain multiple species. Potential species present in these samples could include LiOH, Li_2_CO_3_, and Li_2_O, which have been reported in battery and lithium-mediated nitrogen reduction literature.^11,18,21,43,44^ Interestingly, increasing ethanol concentration appears to greatly increase the intensity of what is ascribed to Li_2_O at around 528 eV. This is reminiscent of XPS data published from our group investigating the impact of electrolyte water^19^ content. Here, a peak at around 528 eV increased with increasing water content. In the case of increasing water content, the assignment of this peak was complicated by the presence of possible copper oxides which would also have a peak at this binding energy.^19^ For the measurements in this work, the SEI was thick enough such that the Mo core levels were not observed in survey spectra for ethanol concentrations from 0 to 34 mM (Figure S7). For the 86 mM ethanol condition, however, there was some evidence for Mo in the survey spectra. However, since the atomic ratio of lithium and oxygen both rose together at 86 mM (Figure 4a), we assume that Mo oxide only makes a small contribution to the peak assigned to Li_2_O. In work by Li and Andersen et al., the addition of trace oxygen in the inlet gas stream was shown to improve Faradaic efficiency toward ammonia. In this work, X-ray diffraction analysis showed the presence of Li_2_O, and the authors stated that the presence of this SEI species resulted in increased SEI resistance, which is beneficial for the balance of reactant transport.^16^ Table S1 shows that the addition of ethanol did not alter the initial water content of the electrolyte, thus eliminating the possibility that the change in the O 1s spectra is simply a result of initial water concentration. Interestingly, Li_2_O was not observed by Steinberg et al.^10^ who used an LiBF_4_ containing electrolyte.

Given the presence of ethanol in the electrolyte, it is highly likely that lithium ethoxide was formed.^28,45^ Lithium ethoxide could be produced by the reaction in eq 3:

$$2\mathrm{Li}+2C_{2}H_{5}\mathrm{OH}\to 2C_{2}H_{5}\mathrm{OLi}+H_{2}$$ 3

However, lithium ethoxide is a highly reactive compound and is likely to further react with trace water to produce either LiOH or Li_2_O via either eq 4 or 5:

$$C_{2}H_{5}\mathrm{OLi}+H_{2}O\to \mathrm{LiOH}+C_{2}H_{5}\mathrm{OH}$$ 4

$$2C_{2}H_{5}\mathrm{OLi}+H_{2}O\to {\mathrm{Li}}_{2}O+2C_{2}H_{5}\mathrm{OH}$$ 5

LiOH can also further react with lithium to form Li_2_O, LiH and H_2_ gas.^43,46^ This could explain the correlation between Li_2_O content in the SEI and ethanol concentration in the electrolyte.

Figure S8c shows the C 1s core level spectra for the five ethanol concentrations considered. All spectra show the presence of organic fluorinated species, as expected from Figure 4b, as well as Li_2_CO_3_ and Li_2_C_2_ which are expected from literature.^10,21^ Figure 4a shows that the relative atomic concentration of carbon decreases with increasing ethanol content. Similar to what was discussed in relation to the F 1s core level, it appears that altering ethanol concentration in the electrolyte also alters the ratio of organic to inorganic species in the SEI. This echoes findings from our group where an increase in salt concentration resulted in more coordinated ionic geometries and a greater proportion of inorganic salt decomposition products in the SEI.^21^ The lithium-ion solvation structure is correlated to the observed lithium plating potential,^47,48^ and we observed an increase in lithium deposition potential with increased salt concentration.^21^ When changing ethanol concentration, as shown in Figure S1e (and Figure 1a), no significant change in lithium deposition potential was observed between 17 and 86 mM EtOH, suggesting that such a change in ethanol concentration does not significantly alter the solvation structure of lithium ions, and therefore reduction in organic species in the SEI are directly due to a chemical change induced by the variation in ethanol content.

Figure S8d shows the variation in the S 2p core level spectra with ethanol concentration. Interestingly, the relative concentration of Li_2_S, a decomposition product of the LiNTf_2_ salt,^49^ increases with increasing ethanol concentration. It is surprising that the relative concentration of this salt decomposition product would be related to the ethanol content. Li_2_O could also be formed from decomposition of the LiNTf_2_ salt,^49^ and the commensurate increase in both Li_2_S and Li_2_O content could suggest that the two are related to salt decomposition. However, the fluorine content of the SEI is also a result of salt decomposition, since neither THF nor ethanol contain fluorine. The relationship between fluorine content and ethanol concentration does not match that of oxygen or sulfur content, which suggests a more complex influence of ethanol on SEI composition than just increasing the proportion of salt decomposition products in the SEI.

It is important to note that the interaction depth of XPS is on the nanometer scale, while the SEIs investigated herein have thicknesses at least 3 orders of magnitude larger. Given the particularly heterogeneous nature of the ethanol free SEI (Figure 2), it is important not to over interpret the impact of surface chemistry. Therefore, titration and ToF-SIMS measurements were undertaken to investigate the impact of ethanol concentration on bulk SEI chemistry.

### Bulk SEI Chemistry

Figure 5 shows the results of titration measurements to quantify species present within the SEI bulk using a method similar to that described by Hobold and co-workers,^31^ Fang and co-workers,^50^ and McShane and co-workers.^18^ Here, the SEI deposits are dissolved in methanol-OD (titration of Li_2_O and LiOH, and total amount of Li species) or deuterated water (titration of Li^0^, LiH, LiF and Li_*x*_N_*y*_H_*z*_), and resultant analytes quantified (Figure S15). The specific detected species are lithium metal (Li^0^), lithium hydride (LiH), lithium oxide (Li_2_O) and hydroxide (LiOH), lithium fluoride (LiF), and mixed lithium–nitrogen-hydrogen species (Li_*x*_N_*y*_H_*z*_). The total amount of all lithium species (including Li^0^ and Li^+^ adducts) within the SEI was also measured by quantitative ^7^Li NMR following SEI dissolution in methanol-OD. In theory, the total amount of lithium species in the SEI should equal the sum of all other titration measurements containing lithium. However, there may be some mismatch originating from residual electrolyte within the SEI, or the presence of lithium containing species not detected via titration, as well as combined error between titration techniques. The quantity of ammonia produced in the electrolyte was also measured. For all these quantifications, three conditions were tested: ethanol free (0 mM), close to optimum ethanol content (34 mM) and too much ethanol (86 mM). The maximum Faradaic efficiency toward ammonia was 15 ± 1% at 0.2 vol % ethanol. This is slightly lower than that reported in Figure 1b, although the trend in Faradaic efficiency with ethanol content remains the same. This is likely due to the use of a new batch of LiNTf_2_ salt, since it is well documented that differences between salt suppliers and salt batches can induce differences in Faradaic efficiency.^21,29^

**Figure 5:**
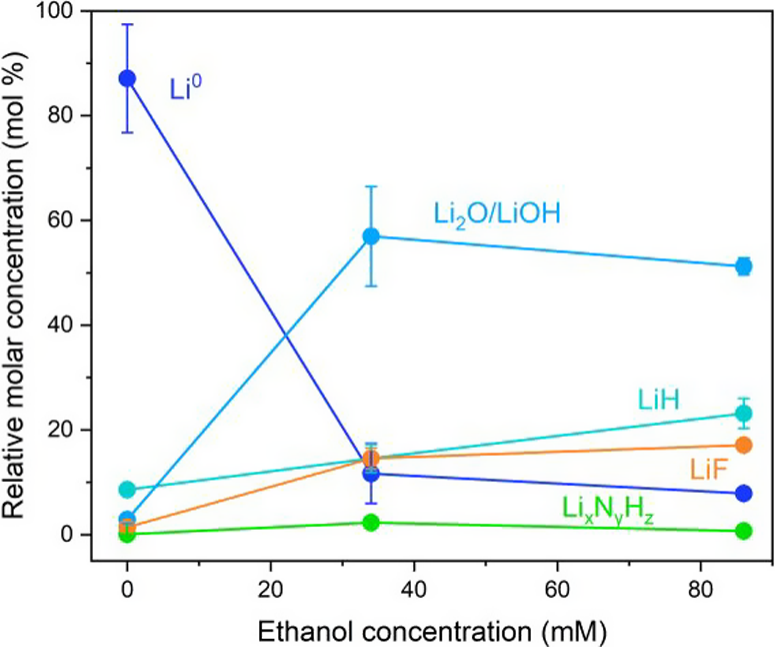
Bulk composition of the SEI following its reactive dissolution post measurement and titration of produced analytes (so-called SEI titration). Taken after −10 C of charge was passed at −2 mA cm^–2^ on a Mo working electrode in a 1 M LiNTf_2_ in THF electrolyte containing different ethanol concentrations. Relative molar concentrations are shown as a percentage of the total quantity of Li^0^, Li_2_O/LiOH, LiH, LiF, and Li_*x*_N_*y*_H_*z*_ measured. Measurement and calculation details can be found in the Supporting Information. Error bars show the standard error in the mean from two separate electrochemical measurements.

As Figure 5 shows, the only sample to contain a significant amount of Li^0^ was that generated in the electrolyte containing no ethanol, supporting the hypothesis that the areas of extremely dark contrast observed in FIB cross sections of the SEI shown in Figure 2b are indeed lithium metal. Furthermore, in situ NMR studies reveal that, although lithium metal can be observed in electrolytes containing ethanol, it quickly disappears upon removal of a reducing potential.^51^ It is likely that any metallic lithium would have reacted to form other species before it could be measured by the post-mortem titration technique.

All three samples contained very small quantities of Li_*x*_N_*y*_H_*z*_, with the most observed for the 34 mM ethanol sample. This sample also generated the most ammonia in the electrolyte (15 ± 1% Faradaic efficiency). It may be that these species originate from N_2_ gas, but isotopically labeled experiments would be required to be certain of their origin. From the ToF-SIMS measurements shown in Figures 6 and S11–S13, although lithium–nitrogen containing species were observed in all three SEI samples, they were found to originate in majority from the LiNTf_2_ salt rather than dinitrogen gas. However, it would be logical to assume that more nitrogenated lithium species would be generated in the sample which obtained the highest Faradaic efficiency toward ammonia. Figure 5 shows that the Li_2–*x*_OH_*x*_ (combined Li_2_O+LiOH) content increases with ethanol content. Consistently with the increase observed by XPS (Figure 4), it may be that this signal is dominated by Li_2_O at higher ethanol concentrations. The XPS results also suggest a greater proportion of LiF at 34 mM ethanol. It is likely that these chemical changes altered the transport balance of lithium ions, nitrogen, and protons through the SEI. LiF and Li_2_O are both poorly ionically conductive,^52^ and so the presence of these species could reduce the diffusivity of lithium ions and protons through the SEI and boost nitrogen reduction Faradaic efficiency.^53^

**Figure 6:**
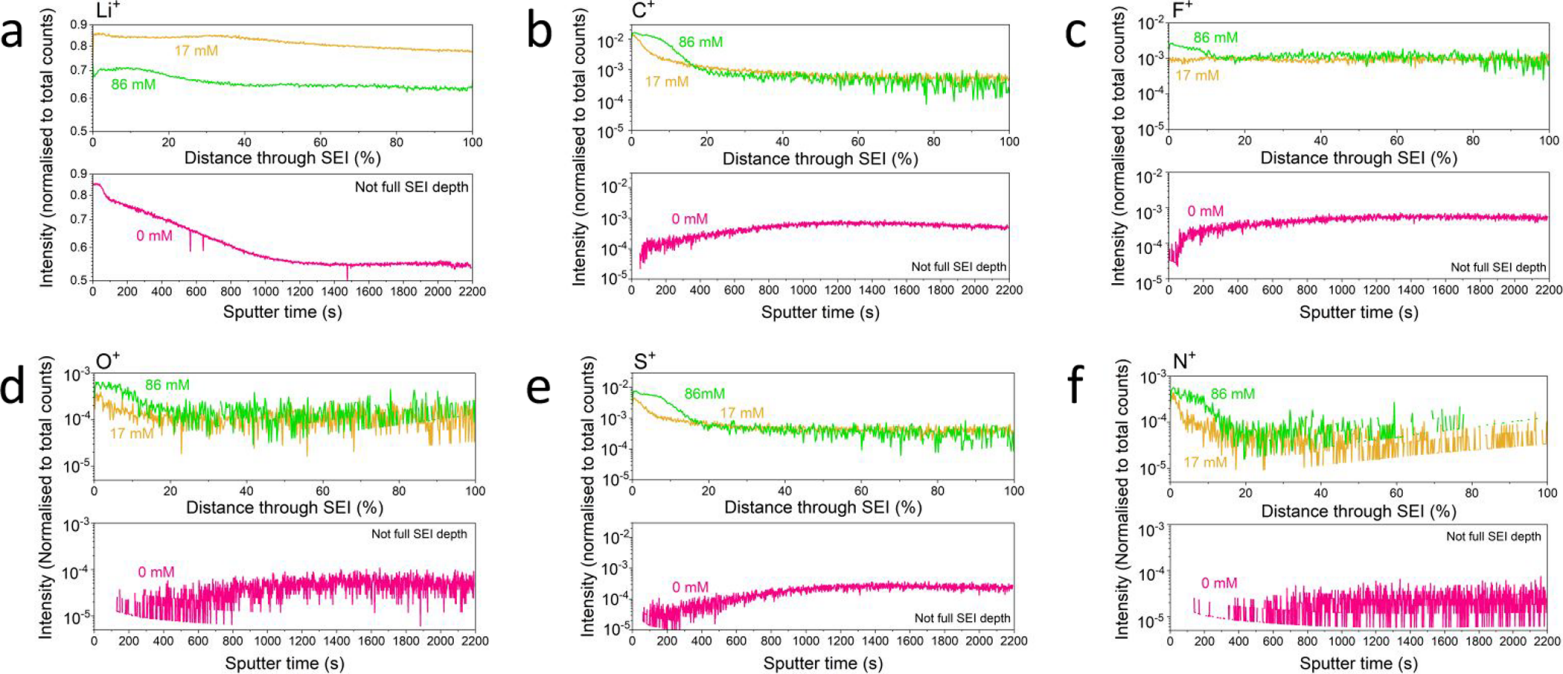
Time of flight secondary ion mass spectrometry traces showing the variation in relative intensity for various fragments of interest through the depth of a solid electrolyte interphase sample. The SEI samples were formed in a 1 M LiNTf_2_ in THF electrolyte containing different ethanol concentrations after the application of −2 mA cm^–2^ on a 1 cm^2^ Mo electrode until −10 C were passed under 1 bar N_2_. The different ethanol concentrations were 0 (pink, 0 vol %), 17 (yellow, 0.1 vol %), and 86 mM (0.5 vol %, green). For the 17 and 86 ethanol samples, the full depth was probed, and so relative depth is shown as a percentage distance through the SEI layer (0% being the surface, 100% being the SEI-electrode interface). For the 0 mM ethanol sample, the SEI layer was too thick to be probed all the way to the electrode interface. These traces are shown with respect to sputter time instead. The fragments of interest are (a) Li^+^, (b) C^+^, (c) F^+^, (d) O^+^, (e) S^+^, and (f) N^+^. Full experimental details and further traces can be found in the SI.

These titration measurements suggest that, similarly to the conclusions of Steinberg et al., the SEI formed in the absence of ethanol is dominated by metallic lithium and prevents access of nitrogen to the active lithium surface. Contrary to the observations of Steinberg et al., however, we observe that the introduction of ethanol increases the amount of Li_2‑x_OH_*x*_ produced, the presence of which likely modulates the ionic conductivity of the SEI. This suggests further interplay between the choice of salt and ethanol concentration, since this work was carried out in LiNTf_2_ and Steinberg et al. used LiBF_4_. Rather than simply disrupting SEI formation to allow for reactant ingress, the addition of ethanol also appears to act as an SEI additive in its own right.

### Time of Flight Secondary Ion Mass Spectrometry (ToF-SIMS)

To gain an understanding of the variation in SEI chemistry with depth, SEI samples were measured using ToF-SIMS and milled using Ar_n_^+^ clusters, since this induces much less sample damage than single ion sputtering.^54–56^ Figure 6 shows the results of ToF-SIMS depth profiling of the 0, 17, and 86 mM ethanol SEI samples. The Li^+^, C^+^, F^+^, O^+^, S^+^, and N^+^ fragments are taken as representative of the total lithium, carbon, fluorine, oxygen, sulfur, and nitrogen content, respectively. The 17 and 86 mM ethanol samples were thin enough for the spectrometer to measure throughout the depth of the SEI sample, but the 0 mM ethanol sample was too thick. Therefore, all the data which could be collected for the 0 mM ethanol sample are presented, while the data are cut off at the point where the Mo surface is determined to be reached for the 17 and 86 mM ethanol samples (see Figure S9). All traces are normalized to the total counts at each data point. It is important to remember that the data collected is not quantitative due to the chemical environment of each fragment altering its intensity, known as the matrix effect.^57^ However, by tracking the relative change in each fragment, we can gain insight into how the chemistry of the SEI varies with depth. Only positive fragments are shown since the intensity was generally higher for the fragments of interest in this mode (see Figure S10 for results for the negative mode).

Figure 6a shows the change in intensity of the Li^+^ fragment with depth through the SEI samples. For the 17 and 86 mM ethanol samples, there is not a significant amount of change in the intensity of the Li^+^ fragment with depth. However, the Li^+^ intensity does decrease for the 0 mM ethanol sample. Figure S11g–i shows an increase in intensity for a Li_2_^+^ fragment with depth for the ethanol containing samples, but not for the ethanol free sample. This could originate from a species such as Li_2_O, which was shown by XPS and titration measurements to be more prevalent in the ethanol containing samples (Figure 4).

Figures 6b–f show the variations in the C^+^, F^+^, O^+^, S^+^, and N^+^ fragments with depth, respectively. Interestingly, they all show very similar depth profiles. This could suggest they share the same primary source. Since the LiNTf_2_ salt is made up of all five elements, the simplest explanation is that these profiles represent the depth to which the NTf_2_^–^ anion can penetrate the SEI layer. This suggests that the penetration depth of the salt increases with ethanol concentration, which suggests that the SEI formed in an electrolyte containing more ethanol will be more permeable to the liquid electrolyte. This also means that the SEI will be more permeable to N_2_ gas and proton carrier species, both of which will be dissolved in the electrolyte. This echoes the views of Steinberg et al.,^10^ where some ethanol is required to disrupt SEI formation and allow nitrogen fixation and protonation to take place. However, an SEI which is too permeable to electrolyte will result in diminished nitrogen reduction efficiency since the hydrogen evolution reaction will dominate with unrestricted access to protons.^15,58,59^ Therefore, it is not simply the addition of ethanol that is important for nitrogen reduction, but the addition of the right concentration of ethanol.

Figure 6b shows the change in intensity of the C^+^ fragment with depth through the SEI samples. While the intensity of the C^+^ fragment decreases sharply with depth for the ethanol containing SEI samples, the C^+^ fragment in the 0 mM ethanol SEI sample initially increases in intensity before plateauing. Figure S10a–c show a similar depth profile for the CF^+^ fragment, which likely originates from salt decomposition, either as a primary decomposition product or from further reactions of the salt decomposition products with the organic solvent. The fact that the C^+^ and CF^+^ traces in the 17 and 86 mM ethanol samples are so well correlated could suggest that the bulk of the carbon is found in fluorinated compounds. A CO_3_^+^ fragment was not observed, but some CO_3_^–^ was observed in the negative mode (see Figure S10). Figures S10 d-f show the variation in intensity with depth for other F-containing fragments. For the ethanol containing samples, the F^+^ trace is more highly correlated to the LiF^+^ rather than the CF^+^ traces, while for the 0 mM ethanol sample, the CF^+^ and LiF^+^ fragments appear more correlated to each other. This could suggest a greater abundance of LiF^+^ than CF^+^ in the ethanol containing samples, and more organic fluorine content in the ethanol free sample, as was observed via XPS (Figure 4).

Figure 6d shows the change in intensity of the O^+^ fragment. Figure S10j–l show the variation in intensity for other O containing fragments. All three samples contain an Li_2_O^+^ fragment. For the 0 mM ethanol sample, the intensity of this fragment is well correlated to the O^+^ intensity, suggesting that the two traces share the same origin. However, for the ethanol containing samples, the Li_2_O^+^ intensity is not correlated to the O^+^ intensity, suggesting they do not share the same origin. It is likely, therefore, that the Li_2_O in the ethanol containing samples originates primarily from ethanol decomposition at the cathode, as proposed in eq 5, while the Li_2_O^+^ trace in the 0 mM ethanol case originates from salt decomposition.

Figure 6f shows the change in intensity of the N^+^ fragment, with Figure S12 showing the variation in intensity for other N containing species, including Li and N containing species. Since these fragments can originate from either salt decomposition or reactions of lithium metal with N_2_ and protons, isotopically labeled measurements with ^15^N_2_ gas were also carried out to confirm their origin. Figure S13 and listing S1 show the experimental setup and arduino code used for these measurements. SEIs generated in 0 and 17 mM ethanol under ^15^N_2_ and 17 mM ethanol under Ar were also analyzed by ToF-SIMS, as shown in Figure S14. No ^15^N containing fragments were observed, suggesting that the N^+^ fragment shown in Figure 6f, and the N containing species shown in Figure S12, originated from salt decomposition.

It is interesting to note that, although the LiNTf_2_ salt can decompose to form ammonia and related intermediates, this does not result in measurable quantities of ammonia in the electrolyte since all Ar blank conditions resulted in no measured ammonia. In addition, given that the 17 mM ethanol condition under ^15^N_2_ did yield ammonia, the ^15^N_2_ must have been able to reach the active surface and become activated. However, any formed species must have decayed to form ammonia prior to SEI characterization. Li_3_N is particularly unstable in the presence of protons,^60^ and is therefore unlikely to remain on the electrode long enough to be detected ex-situ when in small amounts. An in situ, operando, isotope sensitive technique is therefore required to see such species, such as FTIR or synchrotron measurements such as those carried out by Deissler et al.^28^

## Conclusions and Outlook

While ethanol certainly plays a role as a proton donor, its role as an SEI additive may be even more critical. This sentiment has been echoed by others,^10,23^ with the work herein presenting a multipronged SEI characterization approach toward increased understanding of the role of ethanol and fundamental understanding of lithium-mediated nitrogen reduction. From cryo-microscopy studies, we learn that ethanol content has a marked effect on SEI surface and cross-sectional morphology. In the absence of ethanol, the LiNTf_2_ based SEI is extremely thick and inhomogeneous, while ethanol introduction increases SEI homogeneity and reduces thickness. Interestingly, morphology changes are different to the one observed in an LiBF_4_ containing electrolyte,^10^ suggesting a synergistic role of ethanol and salt/solvent in creating the interphase. Possible ‘dead’ lithium was only observed in the ethanol free condition, an observation which was corroborated by SEI titration measurements. Open questions remain around the fate of this supposedly ‘dead’ lithium, which in theory should still be active for nitrogen reduction even without the application of a reducing potential. Therefore, the observation of such deposits post-mortem suggests a kinetic barrier toward nitrogen reduction in the 0 mM ethanol condition. However, the interplay between salt choice and ethanol concentration must also be considered.

Post-mortem XPS analysis reveals a strong dependence of SEI chemistry on ethanol concentration, with the relative fluorine content in the SEI reaching a maximum at the optimal ethanol concentration for nitrogen reduction. As ethanol concentration increases, the SEI becomes more dominated by Li_2_O instead. Titration measurements also show an increase in bulk combined SEI Li_2_O and LiOH content with increasing ethanol concentration. This effect is analogous to that observed when both water^19^ and oxygen^16^ concentrations are increased in the electrolyte and inlet gas stream, respectively. Note that an increase in detected Li_2–*x*_OH_*x*_ counterintuitively coincides with a decrease in thickness in this study. While more Li_2–*x*_OH_*x*_ is detected, the layers formed could be denser and/or free of other species participating in SEI thickness. Additionally, conditions where more Li_2–*x*_OH_*x*_ is detected (more EtOH) could be associated with SEI-etching reactions such as hydrogen evolution, thinning the SEI. Therefore, Li_2–*x*_OH_*x*_ content and SEI thickness are not necessarily bound to correlate. In all cases, it appears that some Li_2–*x*_OH_*x*_ is beneficial for nitrogen reduction, but too much results in reduced Faradaic efficiency.^16,19^ These data suggest that increased LiF content is beneficial for efficient nitrogen reduction, like what has been previously observed.^17,30^ However, the bulk titration data suggests that the best performing SEI is dominated by Li_2_O and LiOH rather than LiF. Furthermore, the similarities between the O 1s XPS spectra reported in the presence of increased ethanol, water^19^ and oxygen content are striking, and should motivate further investigation into the role of Li_2_O and LiOH in fluorinated electrolytes for lithium mediated nitrogen reduction, such as has been carried out in battery research.^31^ Furthermore, the addition of ethanol appears to alter the ratio of organic to inorganic species within the SEI, analogous to changing salt concentration.^21^ Thus, the impact of ethanol on SEI chemistry is complex and nuanced, with an impact both on salt and solvent decomposition products.

Post-mortem ToF-SIMS analysis suggests an increase in the permeability of the SEI to the electrolyte with increased ethanol content, since fragments likely originating from the LiNTf_2_ salt, are observed at a greater relative distance through the SEI. This could corroborate the proposition of Steinberg et al.^10^ that ethanol addition to the electrolyte results in a greater degree of electrolyte inclusion within the SEI. It may be that the degree of SEI electrolyte permeability alters the transport of nitrogen and protons to the lithium active surface, thus altering the balance of reactants and the selectivity toward nitrogen reduction. It appears that some degree of electrolyte inclusion is beneficial for nitrogen reduction, since the ethanol free, ‘dead’ Li-containing, SEI results in no measurable ammonia production. However, the mechanism for reactant transport through the SEI is as-yet uncharacterized. It is likely that a variety of different mechanisms play a role, such as reactant transport through pores, along grain boundaries, or via a Grotthus-like mechanism. Investigation into the mode of reactant transport through the SEI will be the focus of future studies. It is important to note that this work presents a robust workflow to overcome issues in adapting advanced characterization techniques to the sensitive nitrogen reduction SEI, which the reader can refer to when implementing such characterizations in their work. Several challenges remain, notably in achieving higher quality microscopy cross sections of the SEI, which is a work in progress. Microscopy images also lack chemical information for multiple reasons already discussed. While other techniques (XPS, ToF-SIMS, titrations) complement this information, finding methods for combined space-chemical information would be beneficial, and is the topic of future work too.

The primary takeaway of this work is that the optimal nitrogen reduction SEI exists in a narrow ‘Goldilocks’ region, where conditions are just right. This is shown schematically in the TOC Figure. We acknowledge that the intermediate EtOH concentration selected for the characterizations presented here varies slightly between 17, 26, and 34 mM. However, the absolute value for peak ethanol concentration is not as critical as identifying the common features of a well performing SEI in the Goldilocks zone we describe in this work, and compare against extremes of 0 and 86 mM EtOH. Therefore, we propose that the selected EtOH concentrations aggregate remains a representative group capable of capturing this Goldilocks regime of intermediate EtOH concentration. As shown in the TOC figure, the optimum ethanol concentration for nitrogen reduction coincides with an SEI with increased LiF content and intermediate electrolyte permeability, Li_2–*x*_OH_*x*_ content, and thickness. This Goldilocks region is relatively narrow; an increase of only 69 mM ethanol from the optimal concentration of 17 mM results in a Faradaic efficiency loss of approximately 20%. The exact ethanol concentration is not an absolute as a different Goldilocks region likely exists for different electrolyte configurations and operating parameters^20^ (e.g., current density and Li morphology,^17,26,29,61^ Li salt,^30,62^ proton carrier,^22,63^ temperature,^64^ etc.), but the broad SEI properties of moderate thickness and electrolyte permeability, as well as passivation to reactants, will likely be beneficial for nitrogen reduction in any electrolyte. Although beyond the scope of this work, an investigation into the interplay of ethanol concentration and current density on SEI morphology and chemistry would be a valuable next step. While this work provides answers to some key questions, certain mysteries remain. Although it appears that moderate electrolyte permeability by the addition of ethanol does activate the SEI for nitrogen reduction, the exact mechanism of reactant transport remains elusive. Indeed, while ethanol is proposed to act as a proton shuttle,^27^ the formation of ammonia in the absence of ethanol (after SEI formation) has also been observed.^23^ Therefore, it appears that ethanol may not be the only proton carrier in the system. Furthermore, it is likely that SEI properties are dynamic, with changes over time having been observed in various in situ measurements.^11,28,65^ The ability to pinpoint exactly where and when ammonia is produced, as well as the SEI structure at that point, is a critical next step toward improving understanding of the lithium-mediated nitrogen reduction system. Although more challenging to develop, o*perando* studies will be instrumental in probing such transient mechanisms, in conjunction with advanced ex situ characterizations. We trust that the work presented here will benefit the field in both undertaking and interpreting such studies. By fully understanding the optimal SEI properties in a high performing, lithium-based electrolyte, we can look toward the targeted optimization of other chemistries, such as those based on calcium^7^ or, ideally, other less energy intensive options,^20,66^ as well as even an artificial SEI. While lithium is certainly capable of activating nitrogen, it also has an advantage over other chemistries in its ability to make a suitable SEI for nitrogen reduction.^20^

Therefore, while this work provides further evidence that ethanol does not act simply as a proton donor, we can also see that lithium does not act only as a catalyst.

## Methods

### Li_0.5_FePO_4_ Reference Electrode Preparation

Li_0.5_FePO_4_ reference electrodes were prepared according to a procedure previously reported.^67^ Within an Ar-filled glovebox, a Li disc was mounted on stainless steel spacer and spring, placed in the negative case of a coin cell. A 18 mm diameter disc was cut out of commercial LiFePO_4_ sheets (MTI Corp.), then placed in the opposite positive case and covered with a Whatmann glass fiber A separator, which was wetted with 70–100 μL electrolyte (1 M LiNTf_2_ in THF). The cell was closed with a cell crimper. Assembled cell was discharged (delithiation at 1.56 mA g^–1^_LiFePO4_, 0.01C rate), until a cutoff voltage of +3.6 V vs Li. The cell was then left to relax to a potential plateau (+3.425 ± 0.004 V vs Li for LiFePO_4_), stable for days in the coin cell. The as prepared electrode was extracted and cut into 8 mm discs hooked on a Cu wire for use in the electrochemical measurements presented in Figure 1a.

### Electrochemical Cell Preparation

LiNTf_2_, THF, and ethanol were used to make electrolytes of 1 M LiNTf_2_ in THF with varying concentrations of ethanol added (0–86 mM, or 0–0.5 vol %). All materials were used as purchased. The water content was shown not to vary with ethanol concentration, as shown in Table S1. The typical water content prior to electrochemistry was approximately 50 ppm for all ethanol concentrations. In all cases, the working electrode was a 1 cm^2^ Mo foil, the counter electrode was a Pt mesh of geometric area 1 cm^2^, and the pseudoreference was a Pt wire. Electrochemical measurements were also repeated (Figure 1a) using a LiFePO_4_ reference electrode prepared as stated above. 1 cm^2^ Mo working electrodes were connected to a Cu wire current collector. The working electrode was dipped in 4 M HCl and rinsed with EtOH, prior to successive polishing with 400, 1500, and 2500 grit silicon carbide paper to a mirror finish and sonication in ethanol. The Pt mesh counter electrode and Pt wire pseudoreference were flame annealed. The single compartment glass cell was then assembled such that the working and counter electrodes were approximately 1 cm apart with the Pt wire pseudoreference between them. The cell was brought into the Ar atmosphere glovebox and filled with 12 mL electrolyte. A sample of blank electrolyte was taken for ammonia quantification. The cell was connected to a closed gas line. Ar gas was passed through to ensure no leaks. The cell was then presaturated with N_2_ gas for 30 min (flow rate around 5 mL min^–1^). After electrochemistry, the cell was purged with Ar to remove N_2_ and avoid contaminating the glovebox atmosphere. Both Ar and N_2_ were 99.9999% (N6) purity and further purified by commercially available purifiers (NuPure) upstream of the experiment. A PTFE coated magnetic stirrer was used to agitate the electrolyte. After electrochemistry, the cell was disassembled inside the glovebox. The electrolyte volume was measured and sampled for ammonia quantification. All cell components except for the working electrode were boiled in ultrapure water (>18.2 MΩ, Sartorius) for 1 h. The working electrode was either stored inside the glovebox for further characterization or removed and cleaned in 4 M HCl to remove SEI species. All components except for the working electrode were stored in a drying oven at 70 °C. The working electrode was stored in air.

### Electrochemical Testing

All experiments were carried out at ambient temperature and pressure. The cell was allowed to rest at open circuit voltage (OCV) during initial nitrogen purging to ensure a stable OCV. An impedance spectrum was taken to determine the uncompensated resistance which was used to correct for ohmic drop. The impedance of the counter electrode was also taken during this measurement and the uncompensated resistance used to correct the potential of the counter electrode. A linear sweep voltammogram (LSV) was taken until lithium plating is clearly seen. The potential of lithium plating (Li^+^/Li^0^) was determined by fitting a linear regression to the current–voltage plot region where lithium plating is observed. The lithium plating potential was defined as the potential where the linear regression meets the *x*-axis (see Figure S1c). A constant current density of −2 mA cm^–2^ is then applied until −10 C of charge is passed (chronopotentiometry, CP). A second PEIS spectrum was taken after the experiment to ensure that the ohmic drop did not change over the course of an experiment. The first ohmic drop measurement was used to correct the data. See Figure S1.

### Ohmic Drop Determination

An impedance measurement was taken before electrochemistry at open circuit between 200 kHz and 200 mHz at an amplitude of 10 mV. Two measures were taken per frequency with 6 points per decade. The spectrum was fitted using the Z-fit function in EC-Lab software (Biologic) using the Randles circuit as an equivalent circuit. See Figure S1. The ohmic drop was removed from data using Ohm’s Law.

### ^15^N_2_ Gas Recirculation Measurements

To determine the origin of nitrogen containing SEI fragments detected by ToF-SIMS, isotopically labeled measurements were carried out using a home-built gas recirculation pump. The setup was inspired by Andersen et al.^6^ and the gas recirculation pump design was adapted from the work of Nielander et al.^68^ The authors gratefully acknowledge the advice of Dr. Adam Nielander in troubleshooting the pump and design adaptations, as well as the Imperial College Hackspace for their assistance in the design and manufacture of the pump. Figure S13 shows the pump design and gas line setup. See Supporting Information for more details about the pump design. The standard protocol for using the gas recirculation pump is to first purge through with N6 Ar for 20 min at a flow rate of 20 mL min^–1^ to remove impurities from the glovebox atmosphere in the gas headspace in the setup. Then, the gas inlet can be switched to the desired gas and flowed at a rate of 10 mL min^–1^ for 15 min to replace the Ar. After that, the gas line was switched to recirculation mode and the gas pump was activated to flow gas for 30 min in a closed loop. The inlet gas supplies were switched off to prevent loss of expensive isotopically labeled gas. After presaturation, the electrochemical procedure was carried out as normal. After electrochemistry, Ar was purged through the setup for 20 min at a rate of 20 mL min^–1^ and the setup disassembled.

### Ammonia Quantification

The ammonia yield in the electrolyte was quantified by the salicylate colorimetric method as described in the group’s previous papers.^20,21^ The method is repeated here for clarity.

#### Salicylate Reagent Preparation

Alkaline solution: 800 mg of sodium hydroxide was dissolved in 50 mL ultrapure water to obtain 0.4 M NaOH. The solution was stored at 4 °C in the dark with the sodium hypochlorite solution. Just before quantification, NaOH was mixed with the stock sodium hypochlorite solution in a 9:1 ratio to obtain approximately 1% sodium hypochlorite.

Sodium nitroprusside solution: 149 mg of sodium pentacyanonitrosylferrate­(III) dihydrate was dissolved in 10 mL ultrapure water to make a 0.05 M solution. The solution was stored at 4 °C in the dark.

Salicylate (catalyst) solution: 40 g sodium salicylate was dissolved in 50 mL ultrapure water, to which 1 mL of the sodium nitroprusside solution was added. Volume was diluted to 100 mL to yield a solution containing 2.5 M sodium salicylate and 0.5 mM sodium nitroprusside. The solution was stored at 4 °C in the dark.

Sodium salicylate purification: Sometimes, the sodium salicylate was found to have impurities. To remove these, a purification procedure was carried out. 40 g of sodium salicylate was dissolved in 3000 mL ultrapure water. 50 mL of 6 M HCl was added dropwise to form a white precipitate (salicylic acid), which was removed by filtration and washed with ultrapure water. The salicylic acid was dried at 40 °C under vacuum overnight.

Salicylate (catalyst-purified) solution: For every 10 g of salicylic acid, 17.5 mL of 4 M NaOH and 290 μL sodium nitroprusside solution was added. The solution was diluted to 29 mL.

#### Sample Preparation

Immediately after the end of an electrochemistry experiment, 8 samples of electrolyte were collected (volume ranging between 100 and 400 μL depending on predicted ammonia concentration). Prior to the experiment, two aliquots of the same volume of blank electrolyte were also collected. All samples were removed from the glovebox in sealed vials. For every 400 μL of sample, 20 μL of 4 M HCl was added to fix any evolved NH_3_ as NH_4_Cl. The samples were then evaporated in a water bath at between 65 and 70 °C until a dry residue was obtained (approximately 1 h). The standard addition method as described in our previous work^20^ was used to quantify ammonia. Here, successively increasing volumes of a solution of known concentration (250 ppm) of NH_4_Cl in ultrapure water was added to samples to form samples spiked with different NH_4_Cl concentrations. Sample preparation was carried out as follows:

The remaining solids in sample vials were dissolved in 1 mL ultrapure water and added to cuvettes to yield 8 samples postelectrolysis and 2 blank samples. The two blank samples were diluted to 2 mL with more ultrapure water. One of these samples is for ammonia quantification, and the other is for background correction. Four of the postelectrolysis samples were also diluted to 2 mL with ultrapure water. One of these samples is kept for background correction. To the final 4 samples, 20, 30, 40, and 50 μL of the 250 ppm of NH4Cl solution were added. The samples were then diluted to 2 mL with ultrapure water.

560 μL ultrapure water was then added to the two background correction samples. To the other samples, 280 μL of the salicylate catalyst solution was added followed quickly by 280 μL of the alkaline solution. The samples were then left to develop in the dark for 45 min.

### UV–Vis Spectroscopy

Samples were then analyzed by UV–vis absorption spectroscopy between 400 and 900 nm (Figure S2). Figure S2a shows a representative experiment with the spectra obtained for each sample. The difference in absorbance between the maximum (650 nm) and baseline (900 nm) is used to determine the absorbance of each sample. The blank absorbance is subtracted from the postelectrochemistry samples to remove interference from the negligible quantities of background ammonia (likely primarily originating from the ultrapure water and salicylate reagents). A linear regression of the obtained absorbances is then performed (Figure S2b). The concentration of ammonia in the electrolyte corresponds to the negative of the *x*-intercept, or the ratio of the slope (*m*) of the linear regression and its *y*-intercept (*c*).

### Post-Mortem Characterization

Electrodes used for characterization were stored inside the Ar atmosphere glovebox until they could be analyzed.

#### XPS Sample Preparation and Method

XPS samples were rinsed in 0.1 mL THF to remove any dried electrolyte on the surface. While this may have removed some weakly bound species, this method avoids results being confused with electrolyte signals. The samples were loaded into a vacuum transfer module and affixed using a Cu clip. The samples were transferred under vacuum to the XPS system (THERMOFISHER Scientific K-Alpha^+^, monochromate, microfocused Al Kα X-ray source, 400 μm spot size). Base pressure was 2 × 10^–9^ mbar. The flood gun was used for charge compensation. Survey spectra (Figure S7) were taken with a pass energy of 200 eV. Core level spectra were taken with a pass energy of 20 eV. Spectra were charge-corrected to the C–C peak at 284.8 eV. Peak fitting was performed using Thermo Scientific Avantage software. The ‘smart’ background was used. Peak widths were allowed to vary between constraints of 0.5 and at least 2 eV. The Lorentzian–Gaussian mix was allowed to vary between 10 and 40%.

#### ToF-SIMS Sample Preparation and Method

ToF-SIMS samples were heat sealed in moisture barrier bags (RS Components, U.K.) and transported to a different Ar atmosphere glovebox where they were mounted on a back-mount sample holder and loaded into an inert atmosphere transfer suitcase. The samples were then transferred to the spectrometer (TOF.SIMS5 IONTOF GmbH, Münster, Germany) in an Ar atmosphere. The suitcase was opened when the pressure of the loadlock chamber was less than 3 × 10^–5^ mbar. The analysis was performed with a 25 keV Bi^+^ primary beam at 1.2 pA in high current bunched mode to provide high mass resolution. Sample sputtering was carried out using the gas cluster ion beam (GCIB) Ar_*n*_^+^ (*n* > 1100) at 10 nA. This is gentle to minimize sample damage. Sputter area was 500 μm × 500 μm, analysis area was 200 μm × 200 μm. The positive spectrum was found to have a higher yield for the fragments of interest. The full depth of the sample was determined to be the point at which the Mo2+ fragment intensity reached a plateau, suggesting the bulk of the signal was molybdenum metal (Figure S9). The 0 mM sample was too thick to sputter the full depth. Unfortunately, a measurement of crater depth after sputtering was not possible since the samples reacted with moisture in the air upon removal from the spectrometer, and the SEI dissolved away.

#### Microscopy Sample Preparation and Method

SEM samples were imaged under cryogenic conditions using a Thermofischer Scientific Helios Hydra DualBeam FIB-SEM which has a cold stage (Aquilos). The cold stage has a temperature of ∼165 °C when actively cooled by nitrogen gas which has been passed through a liquid nitrogen dewar. A dedicated anticontaminator beneath the pole piece is kept at a colder temperature to the stage (∼−190 °C) to act as a coldfinger. All FIB milling was carried out using a Xe+ Plasma source at 30 kV using a maximum current of 15 nA.

Processes under cryogenic operation are more complex than at ambient temperature. Under ambient conditions, it is trivial to deposit a protection layer on the sample prior to FIB milling. This can help to avoid curtaining, an artifact caused by inhomogeneities in the sample. The protection layer is deposited via a gas injection system, which deposits an organo-metallic precursor gas onto the sample surface which is then decomposed to Pt (or another material) in a carbon matrix. However, under cryogenic conditions, this precursor gas condenses everywhere on the sample and requires ‘curing’ by the electron or ion beams. This process is complex and required optimization which was not yet complete when these measurements were carried out. In an attempt to provide a protection layer, some samples were coated in a layer of Au ex-situ by use of an ultrahigh vacuum sputter deposition system connected to an Ar atmosphere glovebox. In another case, a droplet of THF was drop cast on top of the sample and then frozen under liquid nitrogen. In all cases, curtaining was reduced but not eliminated.

After electrochemical preparation, all SEM samples were cut to size then heat sealed in moisture barrier bags and transported to an N_2_ atmosphere glovebox. The samples were then transported to the FIB-SEM, either quickly through air (if precoated with a sputter deposited layer of Au), or using a FerroVac cryo/vacuum suitcase which can transport samples under both cryogenic and high vacuum conditions.

The slightly different preparation methods used for each sample are summarized below:

Figure S6: All samples were transferred under vacuum from the N_2_ atmosphere glovebox to the SEM-FIB. No protection layer was applied.

Figure 2: <10 μL THF was drop cast on the sample inside the N_2_ atmosphere glovebox prior to freezing in liquid nitrogen. The sample was then transferred at cryogenic temperatures and under vacuum to the SEM-FIB.

Figure 3a,b: Sample was transferred under vacuum from the N_2_ atmosphere glovebox to the SEM-FIB.

Figure 3c: <10 μL THF was drop cast on the sample inside the N_2_ atmosphere glovebox prior to freezing in liquid nitrogen. The sample was then transferred at cryogenic temperatures and under vacuum to the SEM-FIB.

Figure 3d: Sample was coated with 1 μm Au without air exposure prior to transport in a heat-sealed bag under Ar to the N_2_ atmosphere glovebox. The sample was then transferred as fast as possible in air to the FIB-SEM (<10 s air exposure).

#### SEI Titration Measurements

Interphase species were quantified by reactive dissolution of specific SEI components, using the workflow shown in Figure S15. A protic titrant was reacted with electrode deposits post electrolysis, to yield different gas-phase and liquid phase analytes, which can then be quantified using different analytical techniques, described in the Supporting Information.

## Supplementary Material



## Notes

Additional experimental details, materials, and methods, photographs of experimental setups and further characterization data by XPS, and ToF-SIMS and microscopy (PDF)

## Abbreviations

FIB: focused ion beam
FTIR: Fourier transform infrared spectroscopy
GCIB: gas cluster ion beam
NMR: nuclear magnetic resonance
SEI: solid electrolyte interphase
SEM: scanning electron microscopy
THF: tetrahydrofuran
ToF-SIMS: time-of-flight secondary ion mass spectrometry
XPS: X-ray photoelectron spectroscopy
