# Supplementary material for: The Role of Ethanol in Lithium-Mediated Nitrogen Reduction
Source: J Am Chem Soc. 2025 Aug 11;147(33):29687–701. doi: 10.1021/jacs.5c03389 (PMC12371868; doi:10.1021/jacs.5c03389)
Supplement: Supplementary file 1 [file ja5c03389_si_001.pdf]

Supplementary information

The Role of Ethanol in Lithium-Mediated Nitrogen Reduction

Olivia Westhead[‡1], Romain Tort[‡1], James O. Douglas[1], Michele Conroy[1], Bethan J. V. Davies[1], Anna Winiwarter[1], Aishah Faisal[1], Matthew Spry[1], Artem Khobnya[1], Mary P. Ryan[1], Maria-Magdalena Titirici[2], Rhodri Jervis[3,4], Ifan E. L. Stephens*[1].

[1] Department of Materials, Imperial College London, South Kensington, London SW7 2AZ, UK

[2] Department of Chemical Engineering, Imperial College London, South Kensington, London SW7 2AZ, UK

[3] Electrochemical Innovation Lab, Department of Chemical Engineering, University College London, Torrington Place, London WC1E 7JE, UK

[4] Advanced Propulsion Lab, Marshgate, University College London, Stratford, E20 2AE, UK

‡ These authors have contributed equally.

Corresponding author:

*Ifan E. L. Stephens – i.stephens@imperial.ac.uk – Department of Materials, Imperial College London, South Kensington Campus, London SW7 2AZ, United Kingdom

Contents

1. Materials 3
2. Methods 3
   2.1 Electrochemical cell preparation 3
   2.2 Electrochemical testing 4
      2.2.1 Ohmic drop determination 5
   2.3 $^{15}N_2$ gas recirculation measurements 5
   2.4 Ammonia quantification 6
      2.4.1 Salicylate reagent preparation 6
      2.4.2 UV-vis spectroscopy 7
   2.5 Error treatment 8
   2.6 Post-mortem characterisation sample preparation and method 8
      2.6.1 XPS sample preparation and method 8
      2.6.2 ToF-SIMS sample preparation and method 9
      2.6.3 Microscopy sample preparation and method 9
      2.6.4 SEI titration measurements 11
3. Further XPS data and discussion 17
4. Further ToF-SIMS data and discussion 18
5. Further microscopy images and discussion 19

1. Materials

Tetrahydrofuran (anhydrous, ≥99.9%, inhibitor-free), sodium salicylate ($C_6H_4(COONa)(OH)$, ReagentPlus®, ≥99.5%), HCl (30 %, Suprapur®), sodium hydroxide solution (30%, Suprapur) and Methanol-OD (reagent grade, ≥99 atom % D) were purchased from Sigma Aldrich. Lithium bis(trifluoromethanesulfonyl)imide (LiTFSI) (≥99.9%, < 20ppm H_2O) was purchased from Solvionic Inc. Ethanol (≥99.5 %, Extra Dry, absolute, AcroSeal™) was purchased from Thermofischer. Sodium pentacyanonitrosylferrate(III) dihydrate (ACS, 99 – 102 %) was purchased from Alfa Aesar. Sodium hypochlorite (14% Cl2 in aqueous solution, GPR RECTAPUR), sodium hydroxide (pellets, AnalaR NORMAPUR) and molecular sieves (4 Å, GPR RECTAPUR®, sonicated in acetone for 10 min, activated at 300 ^{o}C on a hot plate for 10h, then further dried at 100°C under dynamic vacuum overnight before storage and use inside an Argon glovebox) were purchased from VWR. Platinum mesh (wire diameter 0.1mm, nominal aperture 0.4 mm, purity 99.9%), Platinum wire (diameter 0.5 mm, 99.99%, as drawn), Copper wire (0.5 mm, 99.99%, as drawn), and molybdenum foil (0.125 mm thick, annealed, 99.9%) were purchased from Goodfellow, Cambridge. $LiFePO_4$ (double sided coating on aluminium, 27.0 ± 0.4 mg.cm^{-2}, 150 mAh.g^{-1} capacity) commercial sheets were purchased from MTI Corporation. Single compartment glass cell was custom made by Artistic and Scientific Glassware, Oxford. Purifiers for the Ar and N2 gas lines providing purity levels of H_2O, H_2, CO_2, O_2, CO, nonmethane hydrocarbon (NMHC), CH4, NH3, NOx to < 0.5 ppb were purchased from NuPure. N6 Ar and N6 N2 gas was purchased from BOC. $^{15}N_2$ gas (15N2, 98%+) was purchased from CK Isotopes. Glassware for gas recirculation pump was purchased from Adams & Chittenden Scientific Glass Co-op. 316 stainless steel springs used inside gas recirculation pump were purchased from Lee Spring. PEEK piston was fabricated in house. Magnetic body of piston was made from a PEEK encapsulated NeFeB magnet (V&P Scientific). Microcontroller for gas recirculation pump (Arduino UNO Rev3, ATmega328P) was purchased from Arduino. Dual H-bridge for gas recirculation pump (L298N Dual H-bridge Motor Controller module) was purchased from Amazon.com.

Electrochemistry and electrolyte preparation were carried out in an Ar atmosphere glovebox (MBraun, H2O <0.3 ppm, O2 < 0.3 ppm).

2. Methods

2.1 Electrochemical cell preparation

LiTFSI, THF, and ethanol were used to make electrolytes of 1 M LiTFSI in THF with varying concentrations of ethanol added (0 to 86 mM, or 0 to 0.5 vol %). All materials were used as purchased.

The water content was shown not to vary with ethanol concentration, as shown in table S1. The typical water content prior to electrochemistry was approximately 50 ppm for all ethanol concentrations.

In all cases, the working electrode was a 1 cm^2 Mo foil, the counter electrode was a Pt mesh of geometric area 1 cm^2, and the pseudo-reference was a Pt wire, or a $Li_{0.5}FePO_4$ reference electrode when stated (for the electrochemical measurements displayed in Figure 1a).

1 cm^2 Mo working electrodes were used connected to a Cu wire current collector. The working electrode was dipped in 4 M HCl and rinsed with EtOH, prior to successive polishing with 400, 1500, and 2500 grit silicon carbide paper to a mirror finish and sonication in ethanol. The Pt mesh counter electrode and Pt wire pseudo-reference were flame annealed. The single compartment glass cell was then assembled such that the working and counter electrodes were approximately 1 cm apart with the Pt wire pseudo-reference between them. The cell was brought into the glovebox and filled with 12 ml electrolyte. A sample of blank electrolyte was taken for ammonia quantification. The cell was connected to a closed gas line. Ar gas was passed through to ensure no leaks. The cell was then pre-saturated with N_2 gas at for 30 minutes (flow rate around 5 ml/min). After electrochemistry, the cell is purged with Ar to remove N_2 and avoid contaminating the glovebox atmosphere. Both Ar and N_2 were 99.9999% (N6) purity and further purified by commercially available purifiers upstream of the experiment. A PTFE coated magnetic stirrer was used to agitate the electrolyte. Figure S1a shows an image of the electrochemical cell used.

After electrochemistry, the cell was disassembled inside the glovebox. The electrolyte volume was measured and sampled for ammonia quantification. All cell components except for the working electrode were boiled in ultra-pure (>18.2 MΩ, Sartorius) for one hour. The working electrode was either stored inside the glovebox for further characterisation or removed and cleaned in 4M HCl to remove SEI species. All components except for the working electrode were stored in a drying oven at 70°C. The working electrode was stored in air.

2.2 Electrochemical testing

All experiments were carried out at ambient temperature and pressure.

The cell was allowed to rest at open circuit voltage (OCV) for to ensure a stable OCV. An impedance spectrum was taken to determine the uncompensated resistance which was used to correct for ohmic drop. The impedance of the counter electrode is also taken during this measurement and the uncompensated resistance used to correct the potential of the counter electrode (fig. S1b). A linear sweep voltammogram (LSV) was taken until lithium plating is clearly seen. This is found by extrapolating the line for the lithium plating peak up to the 0 current intersection (fig. S1c). A constant

current density of -2 mA cm^{-2} is then applied until -10 C of charge is passed (chronopotentiometry, CP) (fig. S1d). A second PEIS spectrum was taken after the experiment to ensure that the ohmic drop did not change over the course of an experiment. The first ohmic drop measurement was used to correct the data.

2.2.1 Ohmic drop determination

An impedance measurement was taken before electrochemistry at open circuit potential between 200kHz and 200 mHz at an amplitude of 10 mV. The impedance of the working and counter electrodes were measured simultaneously. 2 measurements were taken per frequency with 6 points per decade. The spectrum was fitted using the Z-fit function in EC-Lab software (Biologic) using the Randles circuit as an equivalent circuit. See Figure S1b for example impedance spectra. The ohmic drop was removed from data using Ohm's Law.

2.3 $^{15}N_2$ gas recirculation measurements

In order to determine the origin of nitrogen containing SEI fragments detected by ToF-SIMS, isotopically labelled measurements were carried out using a home-built gas recirculation pump. The setup was inspired by Andersen et al[1] and the gas recirculation pump design was adapted from the work of Nielander et al[2]. The authors gratefully acknowledge the advice of Dr. Adam Nielander in troubleshooting the pump and design adaptations, as well as the Imperial College Hackspace for their assistance in the design and manufacture of the pump.

Figure S13 shows the pump design and gas line setup. As shown in fig. S2a, the pump consists of a glass body (designed and fabricated by Adams & Chittenden Scientific), a PEEK piston containing an NeFeB magnet, two 316 stainless steel springs, and two solenoids. The solenoids are made up of copper magnet wire wound by hand for approximately 1000 turns around a 3D printed spool. The solenoids are held inside two 3D printed clamps (visible in fig. S13b), which were clamped to a modified retort stand. The solenoids are run using an Arduino microcontroller connected to an H-bridge. A small fan was added to cool the heat sink of the H-bridge. A potentiometer was used to regulate the power input from a 12V, 6A laptop charger, as well as the time between coil polarity switching. The Arduino code used to control the pump was adapted from Nielander et al.[2] and is shown in listing S1. Figure S12c shows the gas line design. Gas can either be purged through the pump directly to exhaust, or recirculated in a closed loop using the pump.

The standard protocol for using the gas recirculation pump is to first purge through with N6 Ar for 20 minutes at a flow rate of 20 ml/min to remove impurities from the glovebox atmosphere in the gas headspace in the setup. Then, the gas inlet can be switched to the desired gas and flowed at a rate of

10 ml/min for 15 min to fill replace the Ar. After that, the gas line was switched to recirculation mode and the gas pump was activated to flow gas for 30 minutes in a closed loop. The inlet gas supplies were switched off to prevent loss of expensive isotopically labelled gas. After presaturation, the electrochemical procedure was carried out as normal. After electrochemistry, Ar was purged through the setup for 20 minutes at a rate of 20 ml/min and the setup disassembled.

2.4 Ammonia quantification

The ammonia yield in the electrolyte was quantified by the salicylate colorimetric method as described in the group's previous papers[3,4]. The method is repeated here for clarity.

2.4.1 Salicylate reagent preparation

Alkaline solution: 800 mg of sodium hydroxide was dissolved in 50 ml ultrapure water to obtain 0.4 M NaOH. The solution was stored at 4°C in the dark with the sodium hypochlorite solution. Just before quantification, NaOH was mixed with the stock sodium hypochlorite solution in a 9:1 ratio to obtain approximately 1% sodium hypochlorite.

Sodium nitroprusside solution: 149 mg of sodium pentacyanonitrosylferrate(III) dihydrate was dissolved in 10 ml ultrapure water to make a 0.05 M solution. The solution was stored at 4°C in the dark.

Salicylate (catalyst) solution: 40g sodium salicylate was dissolved in 50 ml ultrapure water, to which 1 ml of the sodium nitroprusside solution was added. Volume was diluted to 100 ml to yield a solution containing 2.5 M sodium salicylate and 0.5 mM sodium nitroprusside. The solution was stored at 4°C in the dark.

Sodium salicylate purification: Sometimes, the sodium salicylate was found to have impurities. To remove these, a purification procedure was carried out. 40g of sodium salicylate was dissolved in 3000 ml ultrapure water. 50 ml of 6M HCl was added dropwise to form a white precipitate (salicylic acid), which was removed by filtration and washed with ultrapure water. The salicylic acid was dried at 40°C under vacuum overnight.

Salicylate (catalyst - purified) solution: For every 10g of salicylic acid, 17.5 ml of 4M NaOH and 290 ul sodium nitroprusside solution was added. The solution was diluted to 29 ml.

Sample preparation: Immediately after the end of an electrochemistry experiment, 8 samples of electrolyte were collected (volume ranging between 100 and 400 µl depending on predicted ammonia concentration). Prior to the experiment, two aliquots of the same volume of blank electrolyte were

also collected. All samples were removed from the glovebox in sealed vials. For every 400 µl of sample, 20 µl of 4M HCl was added to fix any evolved NH3 as NH_4Cl. The samples were then evaporated in a water bath at between 65 and 70 °C until a dry residue was obtained (approximately 1 hour).

The standard addition method as described in our previous work[3] was used to quantify ammonia. Here, successively increasing volumes of a solution of known concentration (250 ppm) of NH_4Cl in ultrapure water was added to samples to form samples spiked with different NH_4Cl concentrations. Sample preparation was carried out as follows:

Remaining solids in sample vials were dissolved in 1 ml ultrapure water and added to cuvettes to yield 8 samples post-electrolysis and 2 blank samples. The two blank samples were diluted to 2 ml with more ultrapure water. One of these samples is for ammonia quantification, and the other is for background correction. 4 of the post-electrolysis samples were also diluted to 2 ml with ultrapure water. One of these samples is kept for background correction. To the final 4 samples, 20, 30, 40, and 50 µl of the 250 ppm NH4Cl solution were added. The samples were then diluted to 2 ml with ultrapure water.

560 µl ultrapure water was then added to the two background correction samples. To the other samples, 280 µl of the salicylate catalyst solution was added followed quickly by 280 µl of the alkaline solution. The samples were then left to develop in the dark for 45 minutes.

2.4.2 UV-vis spectroscopy

Samples were then analysed by UV-vis absorption spectroscopy between 400 and 900 nm (fig. S3). Fig. S3a shows a representative experiment with the spectra obtained for each sample. The difference in absorbance between the maximum (650 nm) and baseline (900 nm) is used to determine the absorbance of each sample. The blank absorbance is subtracted from the post-electrochemistry samples to remove interference from the negligible quantities of background ammonia (likely primarily originating from the ultrapure water and salicylate reagents). A linear regression of the obtained absorbances is then performed (fig. S3b). The concentration of ammonia in the electrolyte ($[NH_3]$) corresponds to the negative of the x-intercept, or the ratio of the slope (m) of the linear regression and its y-intercept (c):

$$[NH_3] = \frac{m}{c} \qquad \textit{Equation S1}$$

The Faradaic efficiency can then be calculated using:

$$FE(\%) = \frac{3[NH_3]VF}{C} \text{ x } 100\%, \qquad \textit{Equation S2}$$

where V is the electrolyte volume, F is the Faraday constant, and C is the charge passed.

2.5 Error treatment

All errors presented represent the standard error in the mean (s), which is given by

$$s = \sqrt{\frac{\sigma^2}{N-1}}$$

Equation S3

where σ is the standard deviation in N measurements.

2.6 Post-mortem characterisation sample preparation and method

Electrodes used for characterisation were stored inside the Ar atmosphere glovebox until they could be analysed.

2.6.1 XPS sample preparation and method

XPS samples were rinsed in 0.1 ml THF to remove any dried electrolyte on the surface. The decision of whether or not to rinse SEI samples for XPS analysis is something which has been discussed widely in the literature. While rinsing samples can help to remove dried salt to clarify spectra, it can also induce SEI damage and remove weakly bound samples[5,6]. Indeed, some researchers in the lithium-mediated nitrogen reduction field choose not to wash their samples at all to avoid SEI damage[7,8]. In other work, researchers use small volumes of the majority solvent rinse their samples – "a few drops" was used by Steinberg *et al.*[9]. We decided to rinse our samples gently to try to mitigate sample damage, but still remove as much dried salt as possible. While this may have removed some weakly bound species, this method mitigates confusion with electrolyte signals, although some LiTFSI peaks do remain (Figure S8). The samples were loaded into a vacuum transfer module and affixed using a Cu clip. The samples were transferred under vacuum to the XPS system (THERMOFISHER Scientific K-Alpha+, monochromated, microfocused Al Kα X-ray source, 400 µm spot size). Base pressure was $2x10^{-9}$. The flood gun was used for charge compensation. Survey spectra (fig. S4) were taken with a pass energy of 200 eV. Core level spectra were taken with a pass energy of 20 eV. Spectra were charge corrected to the C-C peak at 284.8 eV. Peak fitting was performed using Thermo Scientific™ Avantage™ software. The 'smart' background was used. Peak widths were allowed to vary between constraints of 0.5 and at least 2 eV. The Lorentzian-Gaussian mix was allowed to vary between 10 and 40 %.

In order to strength the observation of the outlier measurement at 26 mM ethanol (fig. 4a), we measured two further points at 22 and 30 mM. These single measurements obtained Faradaic

efficiencies of 28 ± 3 % and 36 ± 4 % respectively. However, given that these were single measurements, the exact obtained Faradaic efficiencies are not reliable indicators of performance. Both obtained Faradaic efficiencies are the same to within error as the repeated 26 mM Faradaic efficiency of 30 ± 3% (n=3), suggesting that through repetition they would fit the trend.

2.6.2 ToF-SIMS sample preparation and method

ToF-SIMS samples were heat sealed in moisture barrier bags (RS Components, United Kingdom) and transported to a different Ar atmosphere glovebox where they were mounted on a back-mount sample holder and loaded into an inert atmosphere transfer suitcase. The samples were then transferred to the spectrometer (TOF.SIMS5 IONTOF GmbH, Münster, Germany) in an Ar atmosphere. The suitcase was opened when the pressure of the loadlock chamber was less than $3x10^{-5}$ mbar. The analysis was performed with a 25 keV Bi^+ primary beam at 1.2 pA in high current bunched mode to provide high mass resolution. Sample sputtering was carried out using the gas cluster ion beam (GCIB) Ar_n^+ (n>1100) at 10 nA. This is gentle to minimise sample damage. Sputter area was 500 µm x 500 µm, analysis area was 200 µm x 200 µm. The positive spectrum was found to have a higher yield for the fragments of interest. The full depth of the sample was determined to be the point at which the Mo_2^+ fragment intensity reached a plateau, suggesting the bulk of the signal was molybdenum metal (fig. S9). No fragment suggesting reactions between the Mo substrate and electrolyte were observed. The 0 mM sample was too thick to sputter the full depth. Unfortunately, a measurement of crater depth after sputtering was not possible since the samples reacted with moisture in the air upon removal from the spectrometer, and the SEI dissolved away.

2.6.3 Microscopy sample preparation and method

SEM samples were imaged under cryogenic conditions using a Thermofischer Scientific Helios Hydra DualBeam FIB-SEM which has a cold stage (Aquilos). The cold stage has a temperature of around -165°C when actively cooled by liquid nitrogen. A dedicated anti-contaminator beneath the pole piece is kept at a colder temperature to the stage to act as a cold finger. All FIB milling was carried out using a Xe^+ Plasma source. The instrument did not have an energy dispersive x-ray spectroscopy (EDS) detector, and so such measurements were not possible, although Li would not be observable due to its low atomic number. Cryogenic methods were required due to the instability of SEI samples under the electron beam under ambient conditions[10–12].

Processes under cryogenic operation are more complex than at ambient temperature. Cryogenic operation is also time limited, since cooling only lasts as long as the liquid nitrogen which cools the stage remains liquid. Under ambient conditions, it is trivial to deposit a protection layer on the sample

prior to FIB milling. This can help to avoid curtaining, an artefact caused by inhomogeneities in the sample. The protection layer is deposited via a gas injection system, which deposits an organo-metallic precursor gas onto the sample surface which is then decomposed to Pt in a carbon matrix. However, under cryogenic conditions, this precursor gas condenses everywhere on the sample and requires 'curing' by the electron or ion beams. This process is complex and required optimisation which was not yet complete when these measurements were carried out. In an attempt to provide a protection layer, some samples were coated in a layer of Au ex-situ by use of an ultra-high vacuum sputter deposition system connected to an Ar atmosphere glovebox. In another case, a droplet of THF was dropcast on top of the sample and then frozen under liquid nitrogen. In all cases, reduced but not removed curtaining was achieved.

All SEM samples were cut to size then heat sealed in moisture barrier bags and transported to an N_2 atmosphere glovebox. The samples were then transported to the FIB-SEM, either quickly through air, or using a FerroVac cryo/vacuum suitcase which can transport samples under both cryogenic and high vacuum conditions.

The slightly different preparation methods used for each sample are summarised below:

Fig S2: All samples were transferred under vacuum from the N_2 atmosphere glovebox to the SEM-FIB. No protection layer was applied.

Fig 2: <10 µl THF was dropcast on the sample inside the N_2 atmosphere glovebox prior to plunge freezing in liquid nitrogen. The sample was then transferred at cryogenic temperatures and under vacuum to the SEM-FIB.

Fig 3 (a and b): Sample was transferred under vacuum from the N_2 atmosphere glovebox to the SEM-FIB

Fig 3 (c): <10 µl THF was dropcast on the sample inside the N_2 atmosphere glovebox prior to plunge freezing in liquid nitrogen. The sample was then transferred at cryogenic temperatures and under vacuum to the SEM-FIB.

Fig 3 (d): Sample was coated with 1 µm Au without air exposure prior to transport in a heat sealed bag under Ar to the N_2 atmosphere glovebox. The sample was then transferred as fast as possible in air to the FIB-SEM (<10s air exposure).

2.6.4 SEI titration measurements

Interphase species (Li^0, Li-ions, LiH, $Li_xN_yH_z$, LiF, Li_2O, LiOH, …) were quantified through the following workflow (Figure S15), using different analytical techniques to monitor each analyte resulting from the protonolysis of the electrode material with a titrant solution.

Sample preparation and protonolysis

Protonolysis of an electrode sample consists in reacting the electrode material with a protic molecule to yield an analyte that is quantifiable. Different interphase species require different titrants to be able to quantitatively estimate their amount. In our case, four nitrogen reduction experiments were performed for each electrolyte composition. Two of the as produced electrode deposit them were dipped in THF to wash off residual electrolyte, and titrated with MeOD, allowing the quantification of Li_2O/LiOH and total amount of Li species in the SEI. The two remaining electrode deposits were not dipped in solvent this time, and titrated with D_2O, enabling the titration of Li^0, LiH, $Li_xN_yH_z$ and LiF. Unfortunately, this means that the titration of Li_2CO_3 was not possible (conversion of CO_2 gas is only quantitative in acidic aqueous titrants). However, Li_2CO_3 is suggested to be a metastable species in lithium metal battery SEIs and suggested to convert relatively fast to LiF and CO_2 in fluorinated electrolytes[13] and so we assume a neglectable amount of Li_2CO_3 in such SEIs. The orthogonality between LiF and Li_2O/LiOH titration originates from the fact that LiF is only soluble in water, and Li_2O/LiOH can only be titrated in non-aqueous media.

Species of Interest	Titrant	Washing Sample?	Reaction (analyte **in bold**)	Analytical Technique
Li	D_2O	No	$\mathrm{Li} + 2\ \mathrm{D}^+ \rightarrow \mathbf{D_2} + \mathrm{Li}^+$	Gas Chromatography
LiH	D_2O	No	$\mathrm{LiH} + \mathrm{D}^+ \rightarrow \mathbf{HD} + \mathrm{Li}^+$	Mass Spectrometry
Li_2O/LiOH	MeOD	Yes	$\mathrm{Li_2O} + 2\ \mathrm{MeOD} \rightarrow \mathbf{D_2O} + 2\ \mathrm{LiOMe}$	Karl Fisher
LiF	D_2O	No	$\mathrm{LiF} + \mathrm{D_2O} \rightarrow \mathbf{F^-}_{\mathbf{aq.}} + \mathrm{Li^+}_{\mathrm{aq.}}$	^{19}F NMR
$Li_xN_yH_z$	D_2O	No	$\mathrm{Li_xN_yH_z} + (3y - z)\mathrm{D}^+ \rightarrow y\ \mathbf{NH_3} + x\ \mathrm{Li}^+$	UV-Vis / Ion Chromatography
Li-ions in interphase	MeOD	Yes	Rough eqn: $\mathrm{Li_xR} + x\ \mathrm{D}^+ \rightarrow x\ \mathbf{Li^+} + x\ \text{"RD"}$	^{7}Li NMR / Ion Chromatography

- For D_2O titration: The electrode sample was taken out of the glovebox in a closed septum vial, and 2 ml of titrant was added using a syringe.
- For MeOD titration: MeOD was stored in the glovebox in a closed septum vial, dried over activated molecular sieves (1/3 vol.) for 3 days to yield a solution with < 20 ppm water. 2 ml was then added to the sample using a syringe, and the vial was taken out.

Gas evolution was monitored visually (bubbles formation), taking less than a minute. The sample was taken to gas phase analysis techniques (Gas Chromatography, Mass Spectrometry). The sample was then stored for >10 h (inside glovebox for MeOD titrations), and only then the liquid phase was analysed (Karl Fisher, NMR, Ion Chromatography, …).

Li^0 and LiH Titration – Gas Chromatography + Mass Spectrometry

A method developed by Meng and coworkers[14] for the titration of Li metal was adapted to the quantification of Li and LiH on the electrodes used in electrolytic ammonia synthesis experiments to estimate the amount of excess metal plated and lithium hydride formed. By adding a deuterated proton source (MeOD) to a certain amount of metal (or metal hydride), D_2 (or HD, respectively) is generated following the reactions in equations S4 and S5. The amount of D_2 and HD altogether can be quantified by gas chromatography, and the ratio between the two can be measured by mass spectrometry to trace back to the amount of Li and LiH present.

$$\mathrm{Li} + \mathrm{D}^{+} \rightarrow \mathrm{Li}^{+} + \frac{\mathbf{1}}{\mathbf{2}}\,\mathbf{D_2} \qquad \textit{Equation S4}$$

$$\mathrm{LiH} + \mathrm{D}^{+} \rightarrow \mathrm{Li}^{+} + \mathbf{HD} \qquad \textit{Equation S5}$$

Gas chromatography calibration for hydrogen (H_2, D_2, HD) signal

All Gas Chromatography experiments were performed using a 8610C Multiple Gas Analyzer #5 GC (TCD-FID-METH) from SRI Instruments. A calibration curve was built by hooking a stock gas cylinder containing 1000 ppm (molar) H_2 in CO_2 to the GC in line analysis mode, sampling several dilutions, and running them through the in-line gas chromatography mode (Figure S16, black squares). Since we operate in headspace mode for electrodes titration, the calibration curve was proofed in different ways. First, it was extended to higher H_2 contents by hydrolysing known amounts of $LiAlH_4$ with water (samples made by making several dilutions of 1 M $LiAlH_4$ in THF), which resulted in a calibration curve with a similar slope (Figure S16, green down triangles). Second, one of the points from the in line H_2 gas calibration was repeated using the headspace mode as follows. An open 42 ml septum vial was sunk in a container of ultrapure water, filled with that water and tilted upside down. A gas line was then brought into the vial, flowing and filling it with a mixture of H_2 1000 ppm (molar) in CO_2 (same gas used for the calibration curve). Once full and saturated, the vial was closed with the septum cap while still in the water. The vial was then transferred to the headspace GC for quantification of H_2 as described below here. The results between flow mode and headspace mode concur nicely (Figure S15, red circle). Thirdly, the calibration was also proofed by running the titration of known amounts of lithium. For this, a standard coin cell was assembled with a 18mm Cu current collector facing a Li metal

counter electrode, separated by a Whatman glass fiber disc and a Celgard separator closer to the Cu current collector (to avoid losing too much Li metal stuck to the glass fiber), all wetted with 100 µl of LP57 electrolyte (1M $LiPF_6$ in EC/DMC). The coin cell was run by passing enough charge to plate 100 µg of Li metal at -250 µA. An identical cell was run in the same conditions, then Li metal was stripped at constant current (-250 µA) to estimate how much Li metal had been plated (coulombic efficiency of 18 ± 2% observed, 2.65 ± 0.02 µmol Li^0 expected). The Cu current collector of the first cell was then isolated and stored in a GC vial for titration. The amount of H_2 detected from this sample is slightly under the expected value although quite close (Figure S16, blue up triangles) – expected signal area: 93 ± 0.6 mV.min, obtained: 85 ± 2.4 mV.min. This underestimation is expected as some of the Li metal may have been lost to the current collector when isolating the electrode of interest, or further reacted with electrolyte before isolating the electrode from the coin cell. Some of the generated H_2 might also have been lost through leaks in the GC vial during titration. The calibration curve was deemed acceptable.

Sample analysis by Gas Chromatography – quantification of all hydrogen (H_2, D_2, HD)

After protonolysis of the electrode sample with 2 ml D_2O, it was transferred to the headspace gas chromatography (GC) device, eluting the gas to quantify the amount of hydrogen (H_2, D_2 and HD altogether) and CO_2 generated, operating at 50 °C during analytes elution. Argon was used as a carrier gas, FID (for CO_2) and TCD (for hydrogen) detector were used, with the first one being fed by H_2 generated by a side H_2 generator. A temperature program was set for the GC to operate at 50 °C for the first 20 min, then ramp up to 120 °C (10 °C.s^{-1}) and hold for 5 min to bake out the column. Using the software PeakSimple, the following sequence was run to sample the vial and elute the gas:

Time (min)	Valve and Action	Description
0	Zero	Start of the analysis
0	Sound	Make a sound to alert the user
0.1	E – ON (Valve3Rotate)	Pressurising headspace vial (+ 8 psi)
0.2	E – OFF (Valve3Rotate)	Stop pressurising
0.3	C – ON	Vents the vial into the sample loop
0.4	C – OFF	Stop venting, sample isolated into sample loop
1.0	B – ON (InjectorVent)	Injecting sample into the GC column
20.0	B – OFF (InjectorVent)	End of injection (Ar only into the column)
20.1	F – ON (Valve2Rotate)	Backflush (removing non-eluted components which may interfere with subsequent runs)
26.0	F – OFF (Valve2Rotate)	Stop Backflush

The area of the H_2 peak at ~2 min was noted and traced back to the amount of hydrogen in the glass vial, using the calibration factor generated from the calibration curve (Figure S16). If hydrogen in argon (or CO_2) behaves as an ideal gas, we have:

$$n_{Hydrogen,GC} = Dil_P \frac{PV_{Hydrogen}}{RT} = Dil_P \frac{PV_{gas}}{RT} vol\%_{Hydrogen} = Dil_P \frac{PV_{gas}}{RT} \frac{Area_{Hydrogen,GC}}{78209.155}$$

Where $Dil_P = \frac{101325}{101325+55158}$ (GC vial gas dilution during pressurisation of 8 psi = 55158 Pa)

$$P = 1\ atm = 101325\ Pa, \quad V_{gas} = \left(42 - V_{titrant,liq}\right) * 10^{-6}\ \ (in\ m^3),$$

$$R = 8.314\ J.K^{-1}.mol^{-1} \quad and \quad T = 20^oC = 293.15\ K$$

Estimation of D_2 to HD ratio by mass spectrometry – metal and hydride deconvolution

The mass spectrometer used for this analysis is a SpectroInlets on-chip mass spectrometer, allowing subsecond detection of gases and volatile species with high sensitivity.[3–5] This is possible due to a unique membrane chip that can support liquid or gas on its topside and a gas sampling volume below which is connected to a mass spectrometer through a capillary. The membrane and capillary are fabricated using semiconductor fabrication techniques and are contained within a thumb-sized silicon chip. The chip allows fast equilibration between a top volume and bottom gas sampling volume. A closed cell sits on top of the silicon chip and has a disc-shaped working volume (9 mm diameter, 100 µm thickness. Both top volume and bottom gas sampling volume are pumped down to a vacuum of ~10 mbar before injecting an analyte in the system.

Once the septum vial was sampled for GC analysis, it was taken out of the GC sampler, and 0.5 ml of that same gas phase was sampled from the vial using a gas tight Hamilton syringe and injected into the closed cell built on the mass spectrometer. Masses 2, 3 and 4 (and any other of interest) were recorded before, during and after the injection of the analyte.

The quantification of D_2 (mass 4), HD (mass 3) and H_2 (mass 2) was by integrating such mass signals and deconvoluting them from the signal originating from other species (titrant vapours, other gases …), using a Python program coded by Dr. Anna Winiwarter, using the Python package Ixdat (https://ixdat.readthedocs.io/en/latest/), where the idea behind this code is described in more details in the PhD Thesis of Dr Søren B. Scott[15].

Calculation of the total amount of Li and LiH

Once the total amount of D_2 + HD combined (GC) and their ratio is measured (MS), the amount of Li and LiH in a sample can be calculated as

$$n_{\mathrm{LiH}} = n_{hydrogen,GC} \cdot \frac{n_{\mathrm{HD}}}{n_{\mathrm{HD+D_2}}} \quad \text{and} \quad n_{\mathrm{Li}} = 2\, n_{hydrogen,GC} \cdot \frac{n_{\mathrm{D_2}}}{n_{\mathrm{HD+D_2}}}$$

Lithium Fluoride (LiF) Titration – ^{19}F-NMR

An electrode sample inside a sealed GC vial was hydrolysed with 2 ml D_2O, effectively dissolving LiF into F^- ions. After gas phase analysis, the GC vial was stored for >10 h in a fume cupboard for water to digest all LiF. 0.5 ml liquid was then transferred to a NMR tube if the titrant was D_2O. The as prepared sample was subjected to the following NMR procedure. The experiments were measured on a *Bruker BioSpin* 400 MHz UltraShield NMR magnet with Avance III HD NanoBay console, running TopSpin3.6.5 and equipped with a z-gradient Prodigy/5mm tuneable probe or similar. The spectra were collected at a frequency of 400 MHz with a spectral width of 10 ppm, and 8196 data points giving an acquisition time of 1.09 s. A relaxation delay of 19 s was employed to make sure the signal fully relaxes, running 16 scans and -122.5 ppm offset. The peak at roughly -122 ppm chemical shift (varies with chemical environment) was then integrated and compared against a calibration curve made by subjecting different amounts of LiF dissolved in D_2O to the same NMR procedure (Figure S17).

Lithium nitride/amide/imide ($Li_xN_yH_z$) titration – ion chromatography

An electrode sample inside a sealed GC vial was reacted using 2 ml D_2O, according to equation S9:

$$\mathrm{Li_xN_yH_z} + \mathrm{x\ MeOD} \rightarrow \mathrm{y}\ \mathbf{NH_{z/y}D_{x/y}} + \mathrm{x\ Li^+} \qquad \textit{Equation S9}$$

After gas phase analysis, the GC vial was stored for >10h inside the glovebox for the titrant to digest all metal nitride/imide/amide and yield ammonia. The amount of ammonia produced was quantified by ion chromatography using the following procedure:

Each sample was eluted using a Metrohm 930 Compact IC Flex instrument, on a Metrosep C Supp 2 column, using 5.0 mM nitric acid and 50 ppm rubidium nitrate aqueous eluent, flowing at 1.0ml.min^{-1} at a pressure of 13-14 MPa, and separated cations were detected on a conductivity detector (baseline conductivity, ~0.2 µS.cm^{-1}). The column was then regenerated by purging with a regenerant solution of 70 mM Na_2CO_3 and 70 mM $NaHCO_3$ in DI water. The area of the peak for the ammonium cation was

compared against a calibration curve, to yield in this case the amount of ammonia produced, which can then be traced back to the amount of lithium nitride/imide/amide using equation S6. Calibration curves were made periodically by preparing known amounts of NH_4OH in water and subjecting to the same calibration curve to ensure accurate quantification.

$$\mathrm{n}_{\mathrm{Li_xN_yH_z}} = \frac{1}{y} n_{\mathrm{NH_3}} \text{ (assuming y = 1 in this case (}Li_3N\text{, }Li_2NH\text{ and }LiNH_2\text{))} \qquad \textit{Equation S6}$$

Lithium oxide/hydroxide (Li_2O/LiOH) Titration – Karl Fischer

An electrode sample was dipped in THF to wash off residual electrolyte and left inside a sealed GC vial. It was then protonolysed using 2 ml dry (< 20 ppm) methanol-OD (MeOD), reacting with Li_2O/LiOH according to equations S7 and S8:

$$\mathrm{Li_2O} + 2\ \mathrm{MeOD} \rightarrow \mathbf{D_2O} + \mathbf{2}\ \mathrm{LiOMe} \qquad \textit{Equation S7}$$

$$\mathrm{LiOH} + \mathrm{MeOD} \rightarrow \mathbf{LiOD}/\mathbf{D_2O} + \mathrm{LiOMe} \qquad \textit{Equation S8}$$

After gas phase analysis, the GC vial was stored for >10h inside the glovebox for methanol to digest all metal oxide/hydroxide in the absence of adventitious water. 1.5 ml of this sample was then transferred to a 7 ml septum vial, and the content analysed by Karl Fisher titration. 3x1 ml of the clean "dry" methanol was analysed alongside it for background subtraction, and amount of water detected is calculated with equation S9:

$$n_{\mathrm{H_2O},KF} = \frac{C_{\mathrm{H_2O},ppm} \cdot V_{spl} \cdot \rho_{\mathrm{MeOD}}}{M_{\mathrm{H_2O}}} \qquad \textit{Equation S9}$$

where $\rho_{\mathrm{MeOD}} = 0.813\ g/ml$ and $M_{\mathrm{H_2O}} = 18\ g/mol$

Note that in theory, the amount of water detected does not directly trace back to the amount of metal oxide or hydroxide detected. The acid titration of lithium carbonate Li_2CO_3 releases CO_2 and water in stoichiometric amounts. However, having not detected any Li_2CO_3 through this method of titration, we assume that all water detected originates from Li_2O/LiOH. A limitation of this method is the inability to discriminate Li_2O from LiOH. Therefore, one must assume whether there is a majority of oxide or hydroxide and approximate it to 100% of that phase. This is not a major issue in battery electrolytes where only trace amounts of protic sources leading to LiOH are present, and it can be approximated to 100% Li_2O. However, this assumption does not hold for Li-mediated ammonia

synthesis electrolytes where protic sources are present and both Li_2O and LiOH were observed, as our XPS data suggests. Alternative characterisation would be needed to quantitatively deconvolute the two.

All Li-ions and metal in the deposits

An electrode sample was dipped in THF to wash off residual electrolyte and left inside a sealed GC vial. It was then protonolysed using 2 ml MeOD, stored for >10 h inside the glovebox to digest all Li species into solvated Li ions. The prepared liquid sample was diluted by a factor of 1/5 in D_2O and transferred to an NMR tube. It was then subjected to the following NMR procedure. The experiments were measured on a *Bruker BioSpin* 400 MHz UltraShield NMR magnet with Avance III HD NanoBay console, running TopSpin3.6.5 and equipped with a z-gradient Prodigy/5mm tuneable probe or similar. The spectra were collected at a frequency of 400 MHz with a spectral width of 50 ppm, and 32768 data points giving an acquisition time of 2.11 s. A relaxation delay of 156.65 s was employed to make sure the signal fully relaxes, running 1 scan and 0 ppm offset. The peak at roughly 0 ppm chemical shift (varies slightly with chemical environment) was then integrated and compared against a calibration curve made by subjecting samples with different amounts of LiCl dissolved in D_2O to the same NMR procedure (Figure S18).

2. Further XPS data and discussion

Figure S7 shows the survey spectra collected for each of the samples. There is evidence of some Si contamination for the 17 mM (0.1 vol %) ethanol sample, which originates from the polishing process and is unlikely to affect nitrogen reduction performance. The 86 mM (0.5 vol %) ethanol sample shows some evidence of the presence of Ca, likely from a salt impurity. However, this Ca impurity has a relative concentration of approximately 0.3 %, so is negligible.

Only the 86 mM (0.5 vol %) sample shows evidence for Mo peaks (Mo 3d and Mo 3p), likely since it is much thinner than the other samples. Since the Mo 3p peaks overlap with the N1s, this leads to inaccuracies in the N1s quantification. However, given the small size of the Mo3d peaks, it is likely that any contribution to the N1s signal is small.

Figure S8 shows the Li 1s (a) and N1s (b) core level spectra. There were very few visible features, so these core levels were simply fitted to obtain the relative elemental concentration.

3. Further ToF-SIMS data and discussion

Figure S10 shows the ToF-SIMS depth profiles for negatively charged secondary ions. The relative intensity of these fragments differs than for the positive spectra; for example, for the positively charged secondar ions, the Li^+ signal is the most intense while for the negatively charged spectra it is the F^- signal. This can be explained by the SIMS equation which relates the sputter yield to the energy of the incident beam and fundamental material properties. This is written

$$I_s^x = I_p C_x S \gamma_x F, \qquad \textit{Equation S11}$$

where I_s^x is the secondary ion current of species x, I_p is the primary ion beam current, C_x is the concentration of species x in the sample, S is the sputter yield, γ_x is the ionisation efficiency of species x and F is the transmission function of the analysis system[16]. Although the intensity of the secondary ion current is directly proportional to the concentration of the species in the sample, the chemical state of the species is very influential on the secondary ion current. This is represented by the S and γ_x terms and is known as the matrix effect. The matrix effect makes it very difficult to extract quantitative information in complex, heterogeneous samples[16] such as the lithium-mediated nitrogen reduction SEI. The γ_x term is what determines the charge of the secondary ion. Its magnitude depends on the ionisation energy or electron affinity of the ion of interest. In general, elements with higher ionisation energies are more likely to form positive ions, while elements with higher electron affinities are more likely to form negative ions. Broadly speaking, elements on the left-hand side of the periodic table are more likely to form positive ions, while elements on the right-hand side are more likely to form negative ions[16]. Therefore, the relative intensity of the elements in the positive and negative modes is different.

For the negative mode, the Mo_2^- signal was too low to be able to confidently assign a point at which the molybdenum-SEI interface had been reached. Therefore, all the data collected is presented.

While a CO_3^+ fragment was not observed in the positive mode, a low intensity CO_3^- fragment was observed in the negative mode in all three samples (fig. S10). This corroborates the XPS and titration data (figs. 4 and 6) showing the presence of lithium carbonate. However, from the titration measurements, the quantity of lithium carbonate produced is likely low.

4. Further microscopy images and discussion

Figure S4 shows some further microscopy images of SEI samples formed in a 0 (a-d) and 26 (e-f) mM ethanol containing electrolyte respectively. All samples were briefly air exposed, which may mean that their chemistry was altered. However, it is unlikely that this air exposure would completely alter their morphology.

Figure S5 shows the surface of the bare Mo electrode. There are some scratches, likely from sample handling. In figure S5b, the FIB cross section shows the grain structure of the molybdenum bulk. As shown in Figure S6, the surface morphology of the 0 mM ethanol SEI was very heterogeneous, with large islands and craters visible across the surface. Figure S4 a and b show cross sections taken on a different 0 mM ethanol sample through one of these types of islands, which was an enormous 80 µm thick (Figure S4a) (in comparison, most battery SEIs are on the order of nanometres thick[17]). Further away from the island (Figure S4b), the SEI was thinner at around 30 µm. Figure S4c shows an area of dark contrast, which may be lithium metal, as was observed in Figure 3. This sample was much more curtained, however, so it is harder to make out fine detail. Figure S4d also shows some beam damage to the sample which occurred even under mild imaging conditions (5 kV, 0.1 nA) and at cryogenic temperatures.

Figures S4e and f show a 27 mM (0.15 vol %) ethanol SEI sample, which was approximately 20 µm thick. Similarly to the sample investigated in Figures 4a and b, this SEI appeared more homogeneous than the 0 mM ethanol SEIs. The thickness of the SEI was also similar to that shown in Figures 4 a and b at approximately 20 µm thick. There were however large voids which were not observed in the cross sections shown in Figures 4 a and b. This does not mean that this sample did not contain any voids; the electrodes had a geometric area of 1 cm^2, and the cross sections are on the µm length scale. The large void in Figure S4f was much more vulnerable to charging than the rest of the cross section, shown by the brighter contrast of the void. This could suggest a different chemistry within the void than the surrounding cross section, but a chemically sensitive technique would be required to probe this.

In general, while there is some variation in SEI morphology between samples, the trend of increased homogeneity and decreased thickness with increasing ethanol concentration remains.

Table S1 A table of the measured water content by Karl Fischer titration of 1 M LiTFSI in THF electrolytes with varying ethanol content before electrochemistry (n-2)

Ethanol concentration (vol %)	Ethanol concentration (mM)	Water concentration (ppm)	Error (ppm)
0	0	52	18
0.1	17	50	7
0.2	26	53	6
0.3	34	51	8
0.4	69	45	7
0.5	86	49	3

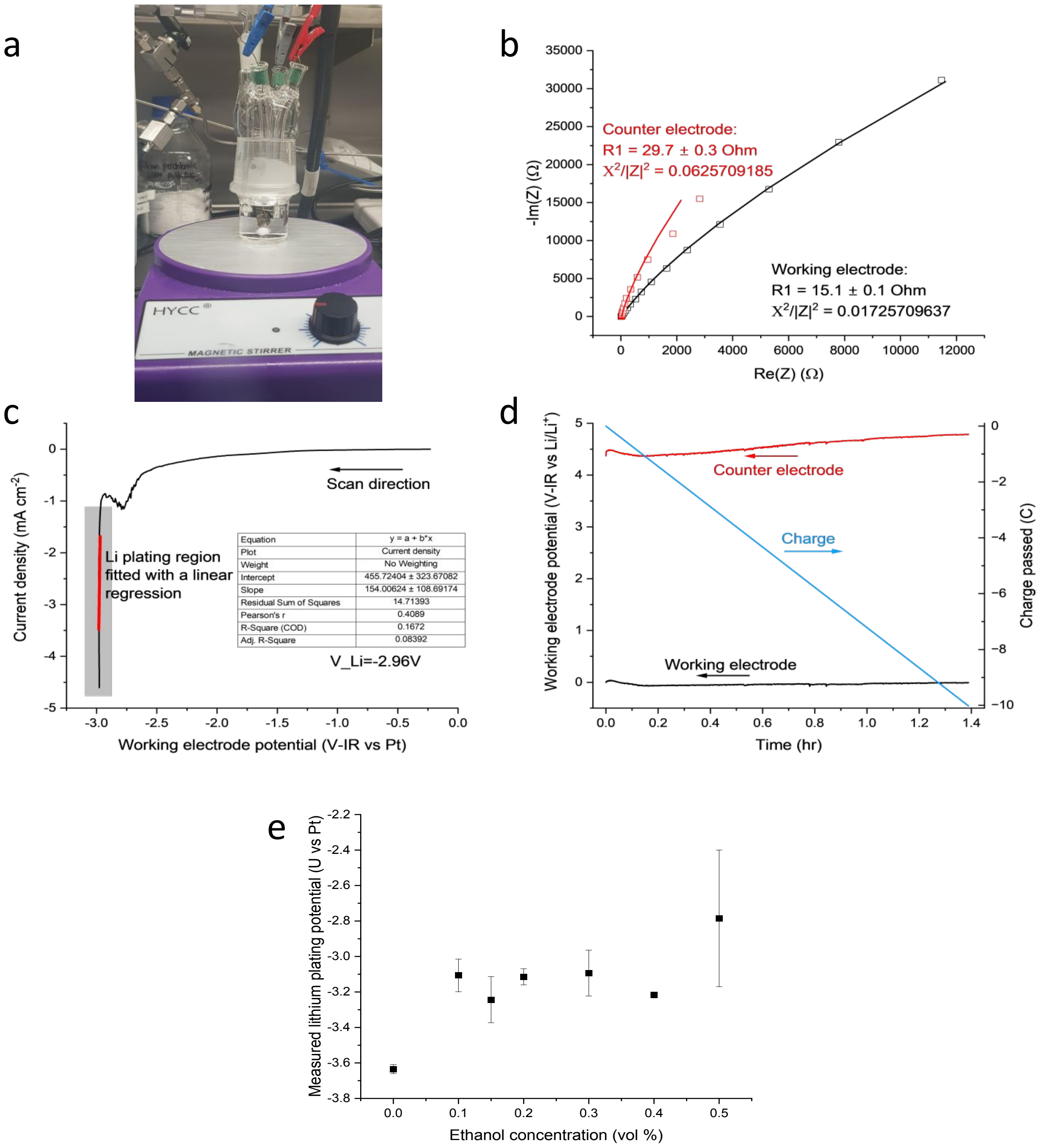

Figure S1 The electrochemical setup and procedure. (a) An image of the glass cell used in these experiments. (b) – (d) example plots showing data from an experiment containing 17 mM (0.1 vol %) ethanol in a 1M LiTFSI in THF electrolyte (Working electrode = Mo foil, counter electrode = Pt mesh, reference electrode = Pt wire) (b) Potentiostatic impedance spectra for the working electrode (black) and counter electrode (red) between 200 kHz and 200 mHz. Both spectra were fitted using the Randles circuit, where R1 represents the ohmic drop. The goodness of the fit is represented by the $\chi^2/|Z|^2$ value. (c) A linear sweep voltammogram from open circuit potential to the lithium plating region. The lithium plating region (highlighted in grey) is fitted using a linear regression (red line) to obtain the

lithium plating potential, which is estimated to be the intercept of this line with the x axis (-2.96 V vs Pt). (d) Chronopotentiometry at -2 mA cm^{-2} until -10 C is passed. The working electrode is close to 0 V vs Li for the entirety of the measurement. (e) The observed variation in lithium plating potential with ethanol concentration. There is essentially no change in the measured lithium plating potential between 17 and 86 mM (0.1 and 0.5 vol %) ethanol, since all variation is within experimental error. For the 0 mM ethanol case, it may be that the plating potential is more negative. The error shown is the standard error in the mean between n=3 separate experiments (n=2 for the 0 mM ethanol case).

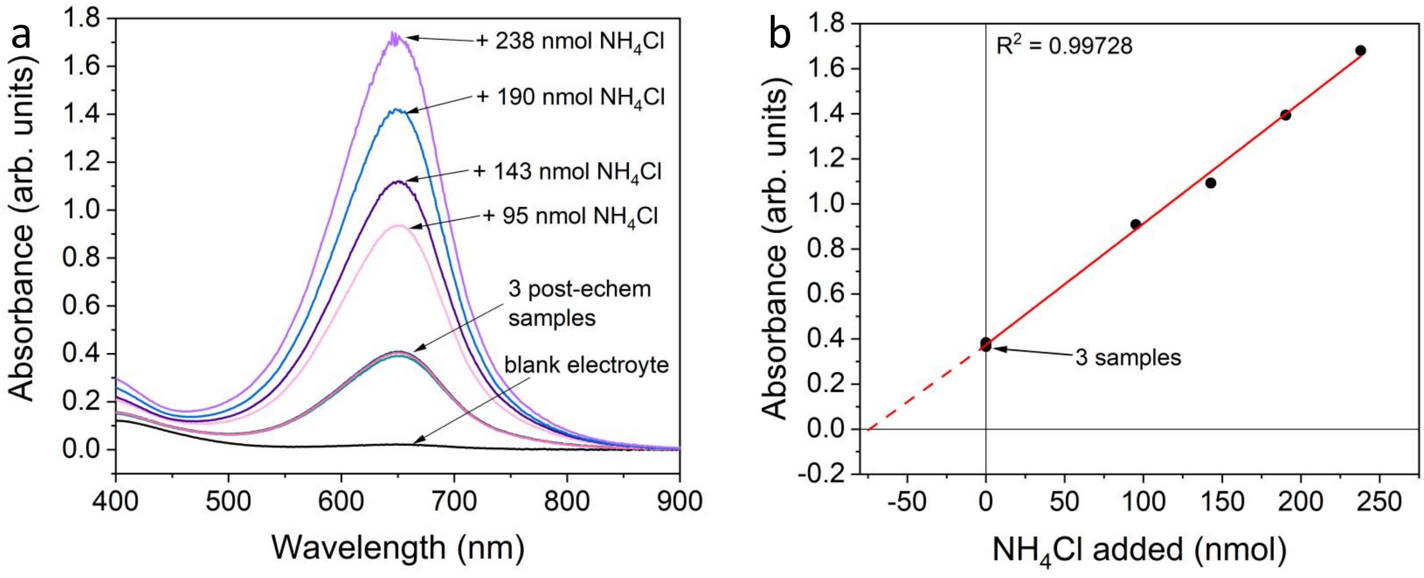

Figure S2 An example of the standard addition method for ammonia quantification. The electrolyte was 1 M LiTFSI in a 99.8:0.2 volume ratio of THF:EtOH. The electrolyte sample volumes were 100 µl. (a) UV spectra generated for a blank electrolyte, 3 samples of electrolyte post electrochemistry, and four further electrolyte samples spiked with NH_4Cl solution. (b) The generated curve of absorbance vs added NH_4Cl content, where the x-intercept represents the amount of ammonia in the unspiked sample.

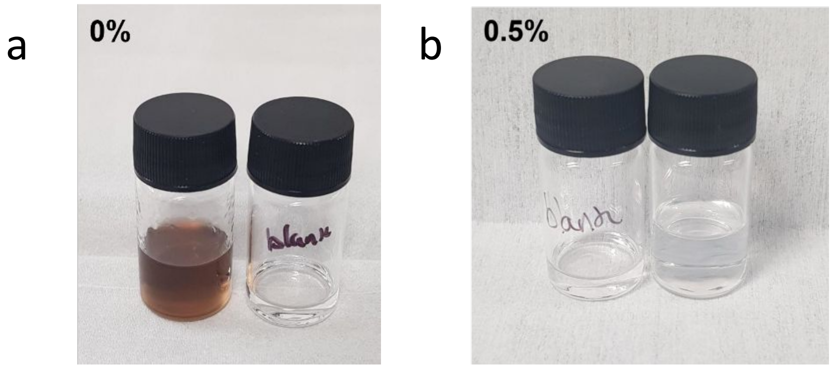

Figure S3 Images of the electrolyte before and after electrochemistry for the 0 mM EtOH (a) and 86 mM (0.5 vol %) (b) conditions. For (a), the blank electrolyte (right) is clear and colourless, while the electrolyte after electrochemistry (left) is severely discoloured. After some time, the electrolyte would eventually turn black and extremely viscous. For (b), the blank electrolyte (left) is also clear and colourless, while the electrolyte after electrochemistry (right) is slightly cloudy. The electrolyte remained stable for longer after electrochemistry, although the slight cloudy feature of the electrolyte suggested some electrolyte decomposition.

For the 0 mM condition, the decomposition products will likely be dominated by poly-THF, which is formed by a ring-opening reaction of the THF. Mygind et al. suggest that the presence of ethanol limits the production of poly-THF through the production of 2-ethoxytetrahydrofuran[18], a decomposition product also observed by Du and coworkers[19], which could explain the difference in electrolyte discolouration between the ethanol containing and the 0 mM conditions.

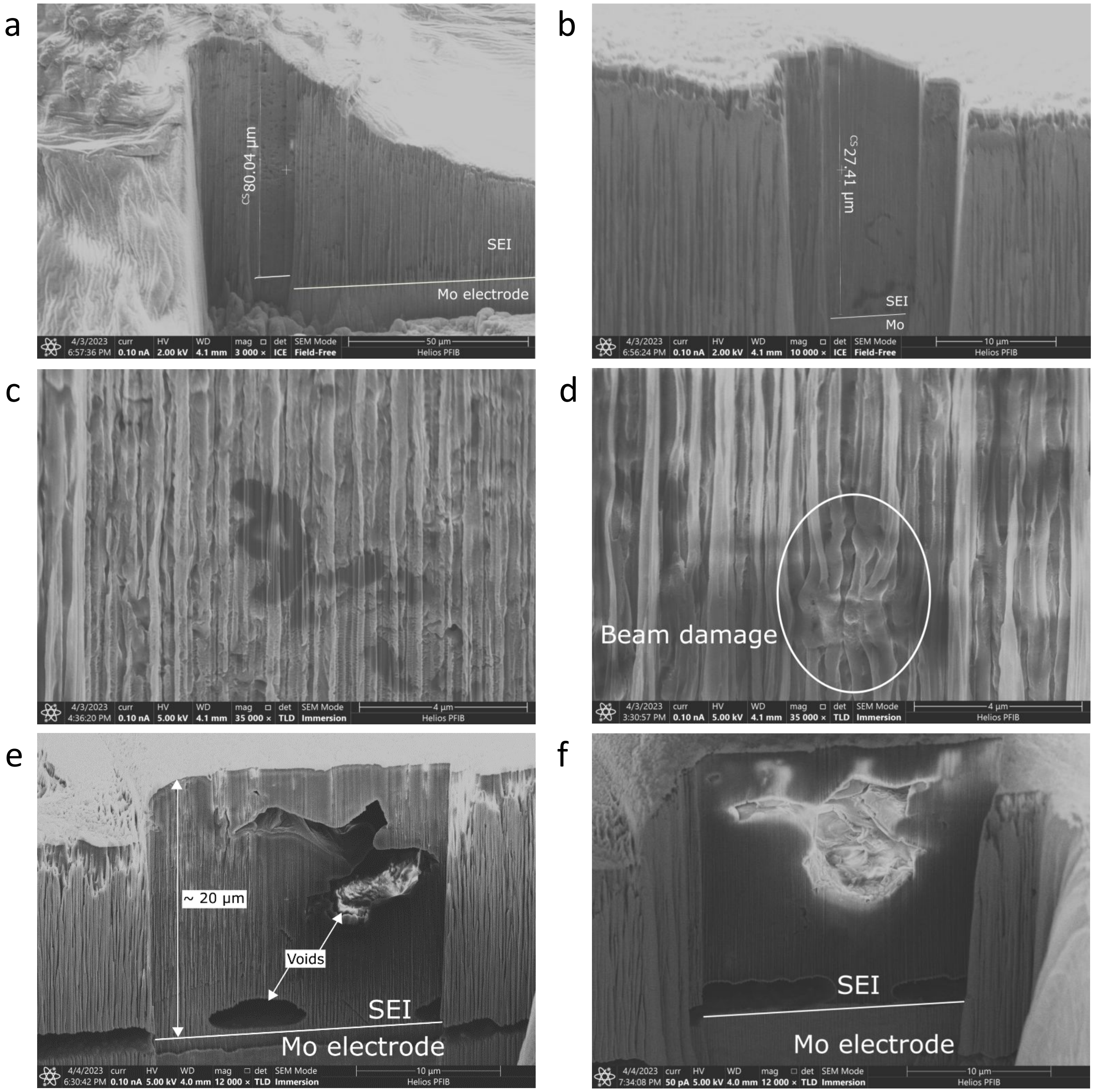

Figure S4 Further microscopy images of SEI samples formed in a 0 (a-d) and a 17 mM (0.15 vol %) ethanol (e-f) electrolyte. The sample imaged in (a-d) was sputter coated in 1 µm Au without exposure to air, but was briefly air exposed upon transfer to the microscope. The sample imaged in (e-f) was not precoated and was air exposed briefly upon transfer to the microscope. The two samples imaged here were formed in separate experiments to those shown in the Figures 2 and 3.

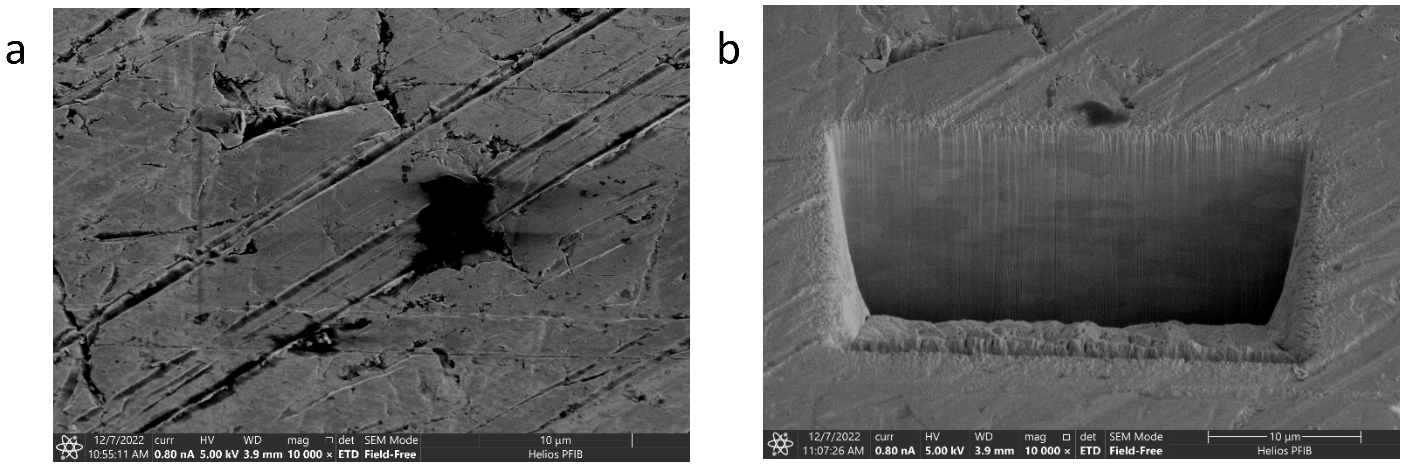

Figure S5 SEM images of the bare molybdenum electrode surface (a) and an FIB cross section of the bare molybdenum electrode (b).

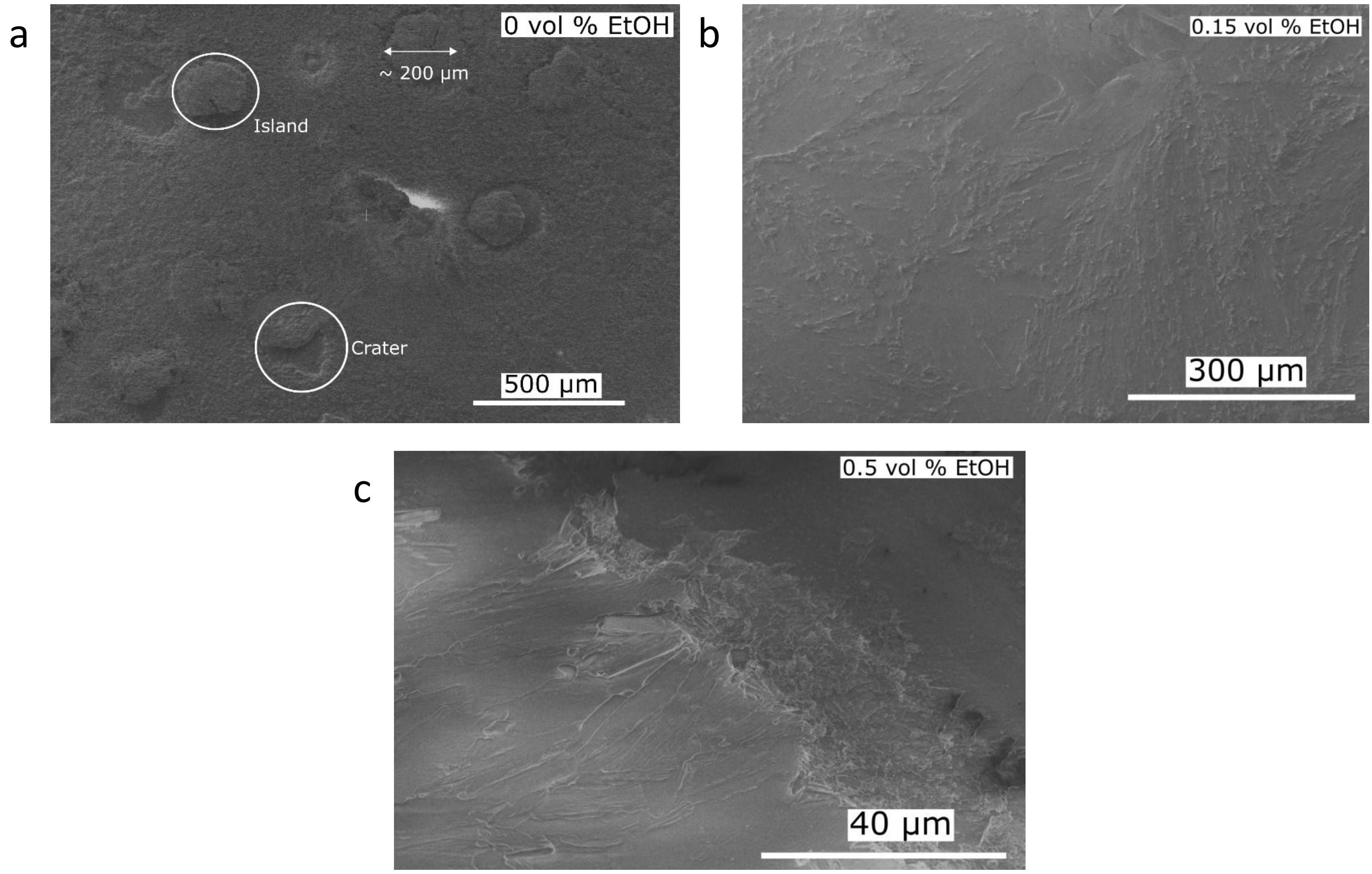

Figure S6: Scanning electron microscopy micrographs of the surface of the Solid Electrolyte Interphase (SEI) layers generated after -10 C was passed at a constant current of -2 mA cm-2 on a Mo working electrode in (a) 0 (b) 17 mM (0.15 vol %), and (c) 86 mM (0.5 vol %) ethanol electrolytes (1 M $LiNTf_2$, THF as majority solvent). Images were taken under cryogenic conditions (approximately -170°C). Samples were all transferred under vacuum from an N_2 glovebox to the microscope.

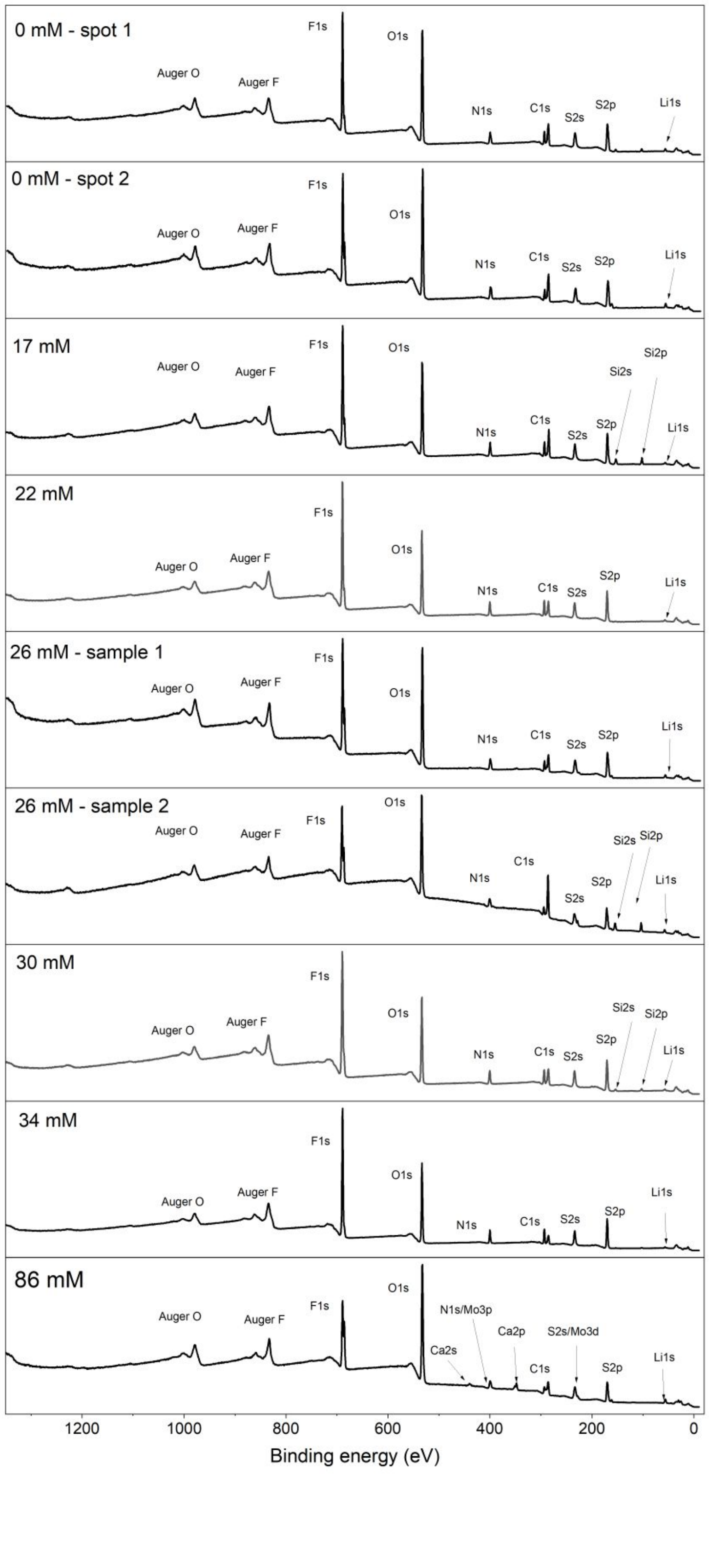

Figure S7 XPS survey spectra for the measurements shown in figure 4. Survey spectra for additional measurements used to calculate average atomic concentrations shown in figure 4a also shown (0 mM

spot 2 and26 mM sample 2), as well as further measurements used to calculate further atomic concentration data points (22 mM and 30 mM).

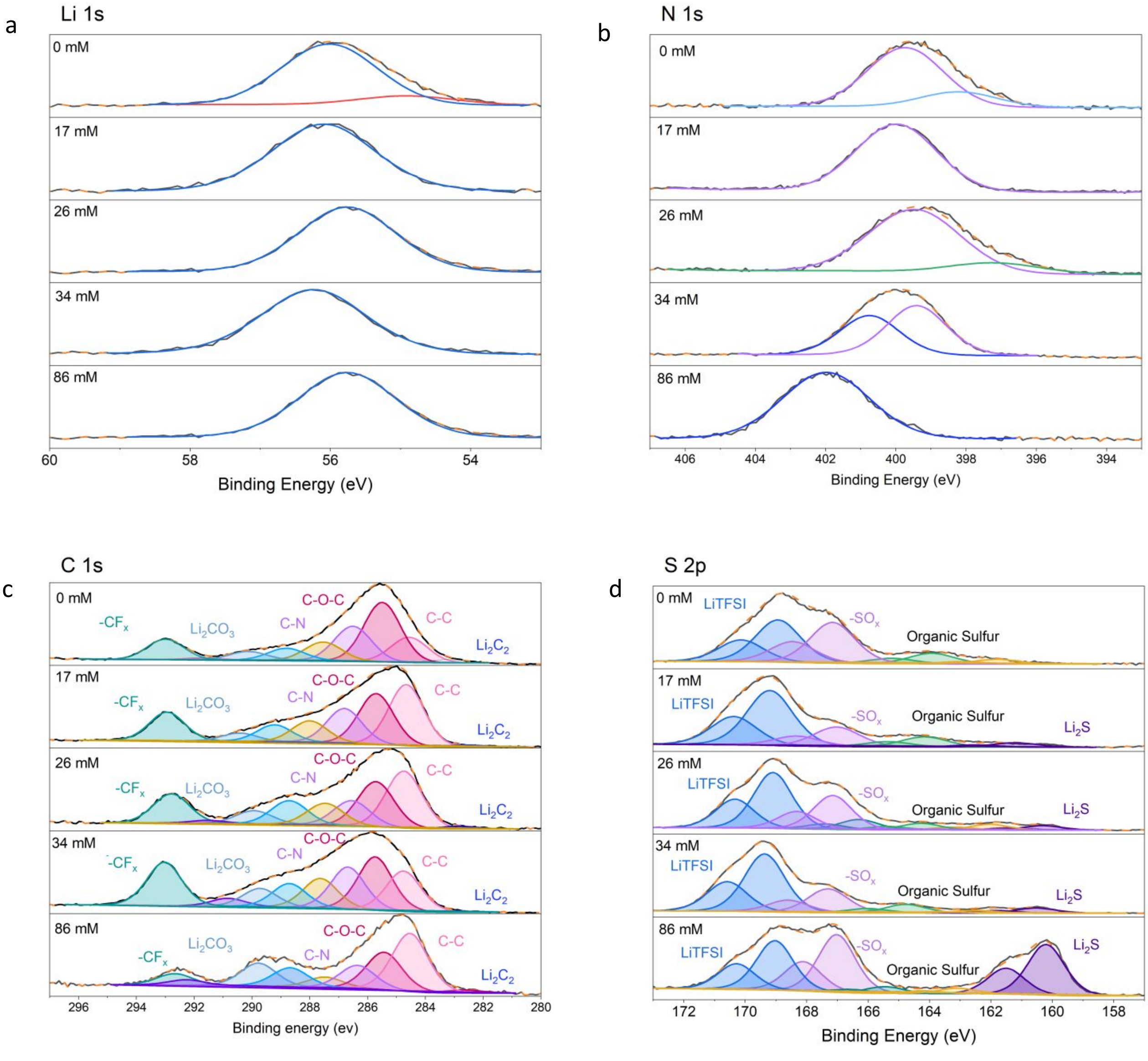

Figure S8 (a) Li 1s, (b) N 1s, (c) C1s and (d) S2p core level spectra for the measurements shown in figure 4.

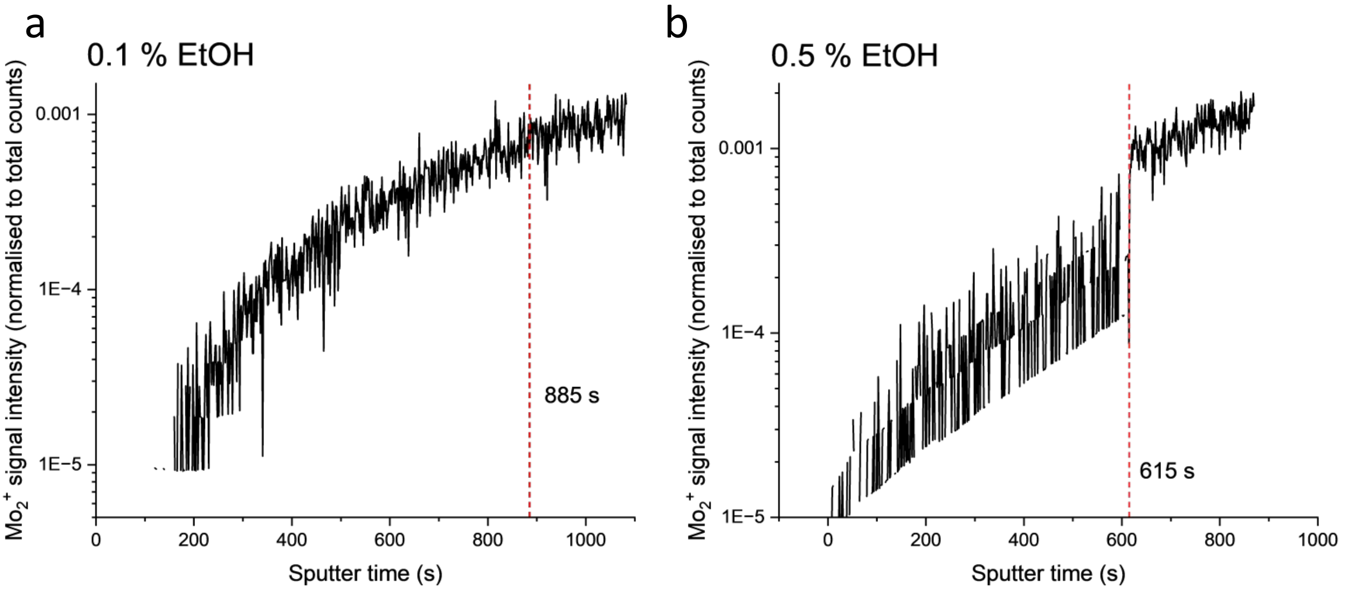

Figure S9 ToF-SIMS Mo_2^+ fragment intensity variation with sputter time for the (a) 17 mM (0.1 vol %) and (b) 86 mM (0.5 vol %) ethanol samples. The red line indicates the time at which the Mo surface is said to have been reached.

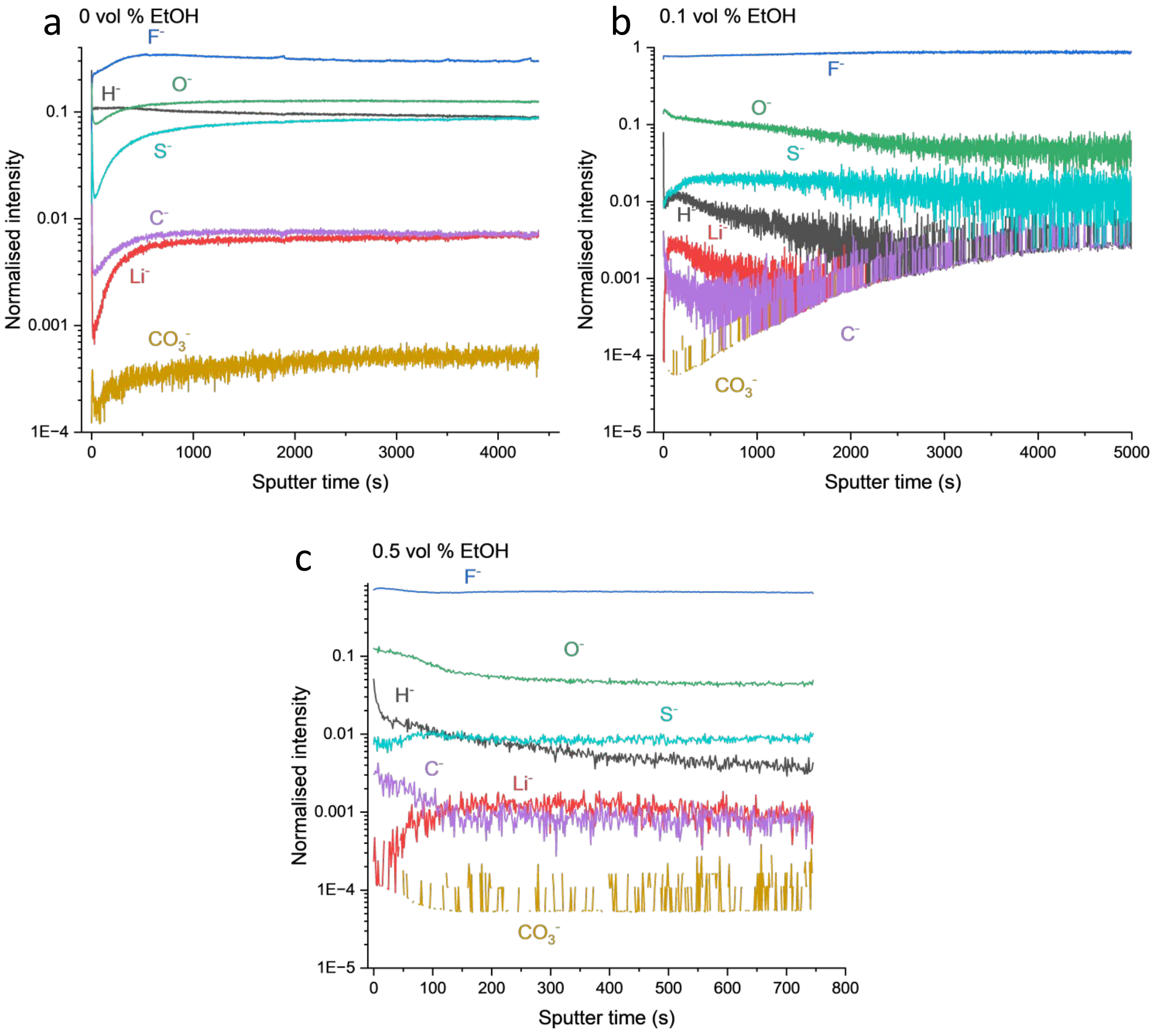

Figure S10 ToF-SIMS traces obtained for the negatively charged secondary ions for the (a) 0, (b) 17 mM (0.1 vol %), and (c) 86 mM (0.5 vol %) ethanol SEI samples

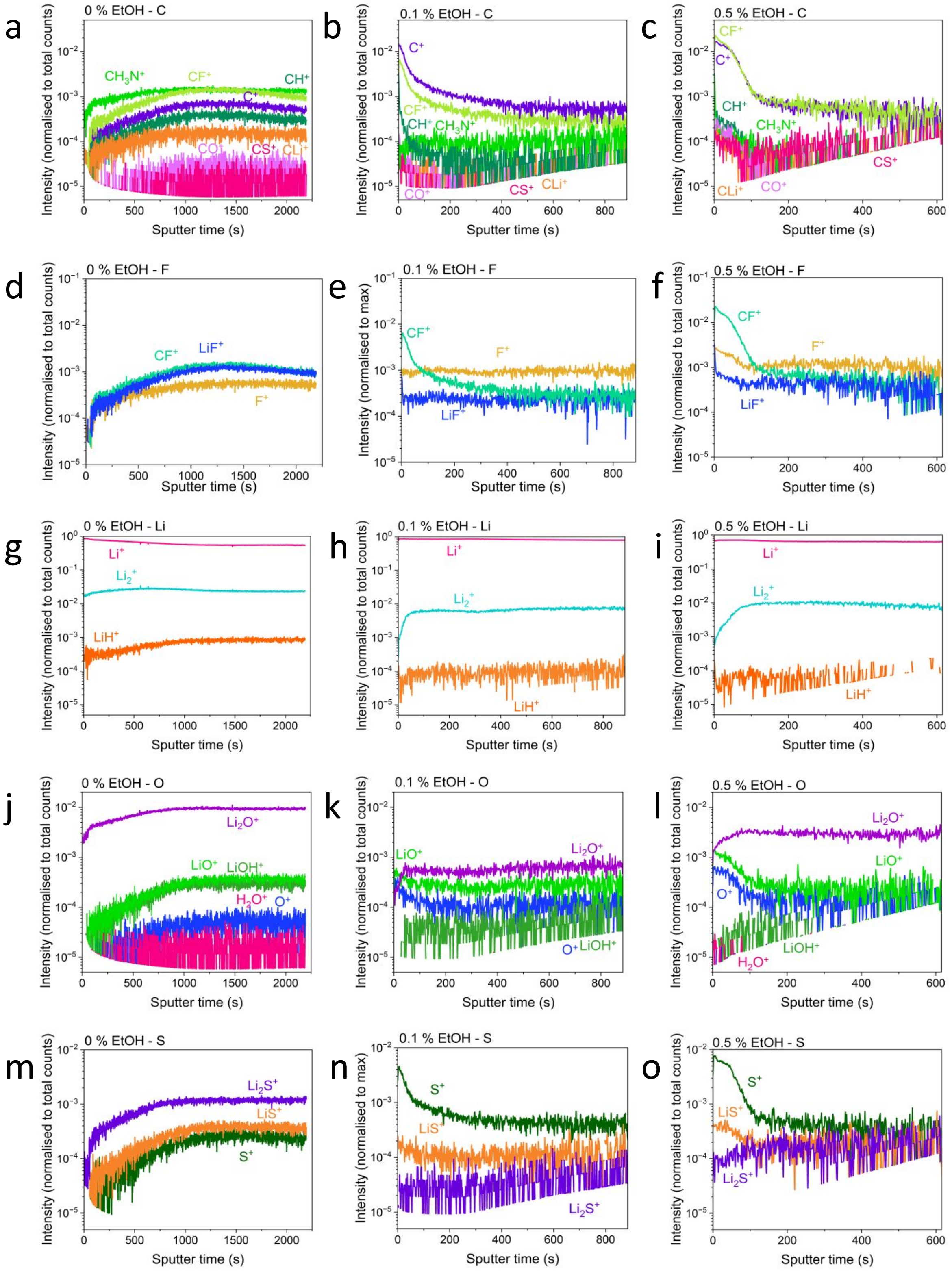

Figure S11 Time of Flight Secondary Ion Mass Spectrometry traces of Solid Electrolyte Interphase (SEI) samples formed in a 1 M LiNf2 in THF electrolyte with either 0, 17, or 86 mM (0, 0.1, or 0.5 vol %) ethanol (left to right) after passing -10 C at -2 mA cm^{-2} on a Mo electrode under 1 bar N_2. For the 17 and 86 mM (0.1 and 0.5 vol %) ethanol samples, traces are cut off at the sputter time at which it was determined the Mo substrate had been reached (see SI). For the 0 mM ethanol sample, the SEI was

too thick to sputter through the full depth, and so all the data which was collected is shown. Depth profiles were obtained using A_n^+ clusters, which induce much less damage than single ion sputtering. (a-c) C containing positive fragments, (d-f) F containing positive fragments, (g-i) Li containing positive fragments, (j-l) O containing positive fragments, (m-o) S containing positive fragments.

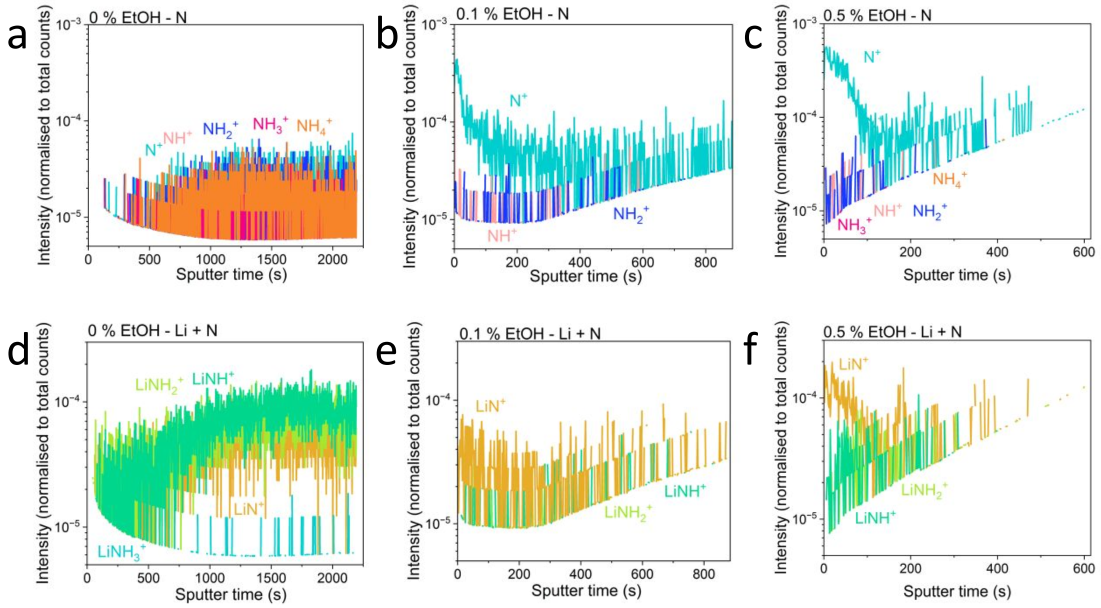

Figure S12 Time of Flight Secondary Ion Mass Spectrometry traces of Solid Electrolyte Interphase (SEI) samples formed in a 1 M LiNf2 in THF electrolyte with either 0, 17 or 86 mM (0, 0.1, or 0.5 vol %) ethanol (left to right) after passing -10 C at -2 mA cm^{-2} on a Mo electrode under 1 bar N_2. N_2. For the 17 and 86 mM (0.1 and 0.5 vol %) ethanol samples, traces are cut off at the sputter time at which it was determined the Mo substrate had been reached (see SI). For the 0 mM ethanol sample, the SEI was too thick to sputter through the full depth, and so all the data which was collected is shown. Depth profiles were obtained using A_n^+ clusters, which induce much less damage than single ion sputtering. (a-c) N containing positive fragments, (d-f) Li and N containing positive fragments.

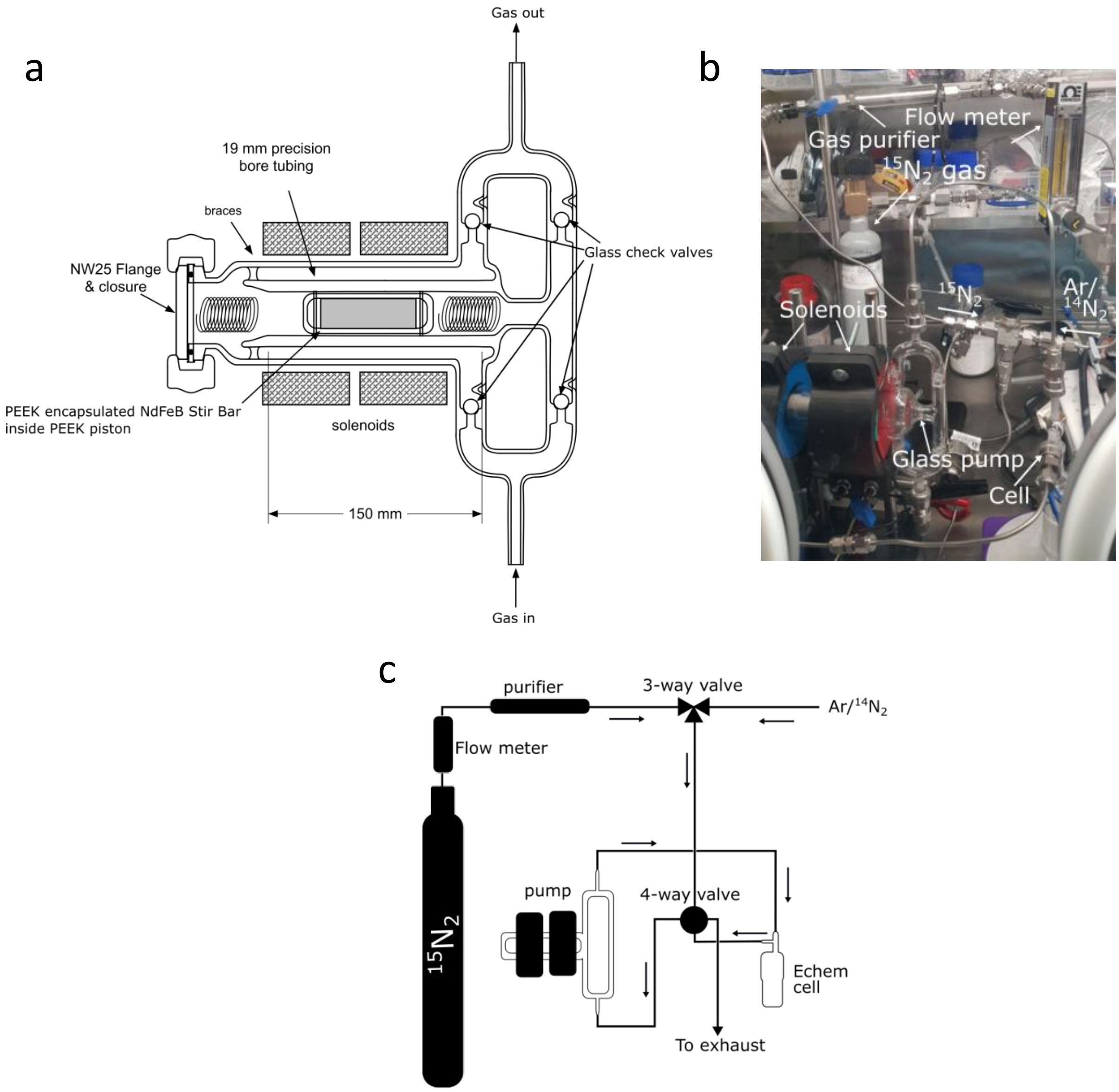

Figure S13 Homemade gas recycling setup for $^{15}N_2$ measurements. (a) A diagram of the glass body gas recirculation pump. Diagram adapted from a technical drawing provided by Adams & Chittenden Scientific Glass. Gas enters through the bottom inlet and is bumped through to the top outlet by the motion of the magnetic piston, which is a PEEK encapsulated NeFeB stir bar encased inside a homemade PEEK piston. The piston is moved by the polarity switching of two homemade solenoids, and two 316 stainless steel springs reduce the piston impact at each end of the pump. The pump is closed by a stainless steel NW25 flange and closure. (b) An image of the $^{15}N_2$ setup within the Ar atmosphere glovebox. (c) A diagram of the recirculation setup. A 3-way valve allows the user to choose between $^{15}N_2$ or Ar or $^{14}N_2$. A 4-way valve then allows the user to choose between purging mode, where the gas passes through the pump and electrochemical cell and goes straight to exhaust, or recirculation mode when the gas travels between the electrochemical cell and pump in a closed loop.

```
// set all the solenoid control pins to outputs
#define enable_solenoid1 10 //define solenoid1 enable as pin 10.
#define enable_solenoid2 5 //define solenoid2 enable as pin 5.
#define in1 9 //pin D9 of arduino, to control H bridge of solenoid 1
#define in2 8 //pin D8 of arduino, to control H bridge of solenoid 1
#define in3 7 //pin D7 of arduino, to control H bridge of solenoid 2
#define in4 6 //pin D6 of arduino, to control H bridge of solenoid 2

const int analogInPin = A0;  // Analog input pin that the potentiometer is attached to

int sensorValue = 0;        // initialise value read from the potentiometer
int power = 0;  // initialise power supplied to coils
int delaytime = 0;  // initialise piston travel time in milliseconds (delay between the two solenoids)

void setup()
{
 // All solenoid H bridge control pins are outputs
 pinMode(enable_solenoid1, OUTPUT); //Feed with potentiometer to control power/speed of soleoids
 pinMode(enable_solenoid2, OUTPUT); //Feed with potentiometer to control power/speed of soleoids
 pinMode(in1, OUTPUT); //digital output pin from arduino to control H bridge
 pinMode(in2, OUTPUT); //digital output pin from arduino to control H bridge
 pinMode(in3, OUTPUT); //digital output pin from arduino to control H bridge
 pinMode(in4, OUTPUT); //digital output pin from arduino to control H bridge
 Serial.begin(9600); // initialize the serial communication
}
void loop()  // function to read the analog in value of the potentiometer and run the pump:
{
    sensorValue = analogRead(analogInPin);
    // map it to the range of the power and delay time:
    power = map(sensorValue, 0, 1023, 200, 255); //255 is 100% duty cycle
    delaytime = map(sensorValue, 0, 1023, 800, 250);
    oscillate_piston(); //function to run solenoids
}
```

```
void oscillate_piston() //define function to run solenoids
{
  delay(delaytime); //wait time set by potentiometer
// turn on solenoid1 forward, piston to the left
 analogWrite(enable_solenoid1, power); //set power of solenoid to what is read in from potentiometer
 digitalWrite(in1, HIGH);
 digitalWrite(in2, LOW);
 // turn on solenoid2 reverse
 analogWrite(enable_solenoid2, power); //set power of solenoid to what is read in from potentiometer in the reverse direction
 digitalWrite(in3, HIGH);
 digitalWrite(in4, LOW);

 // switch off coils
delay(delaytime);

 // inverse of previous push-pull combination.
 // turn on solenoid1 reverse piston to the right
 analogWrite(enable_solenoid1, power);
 digitalWrite(in1, LOW);
 digitalWrite(in2, HIGH);
 // turn on solenoid2 forward
 analogWrite(enable_solenoid2, power);
 digitalWrite(in3, LOW);
 digitalWrite(in4, HIGH);
}
```

Listing S1 Arduino code used to run the recirculation pump. Adapted from Nielander et al.[2]

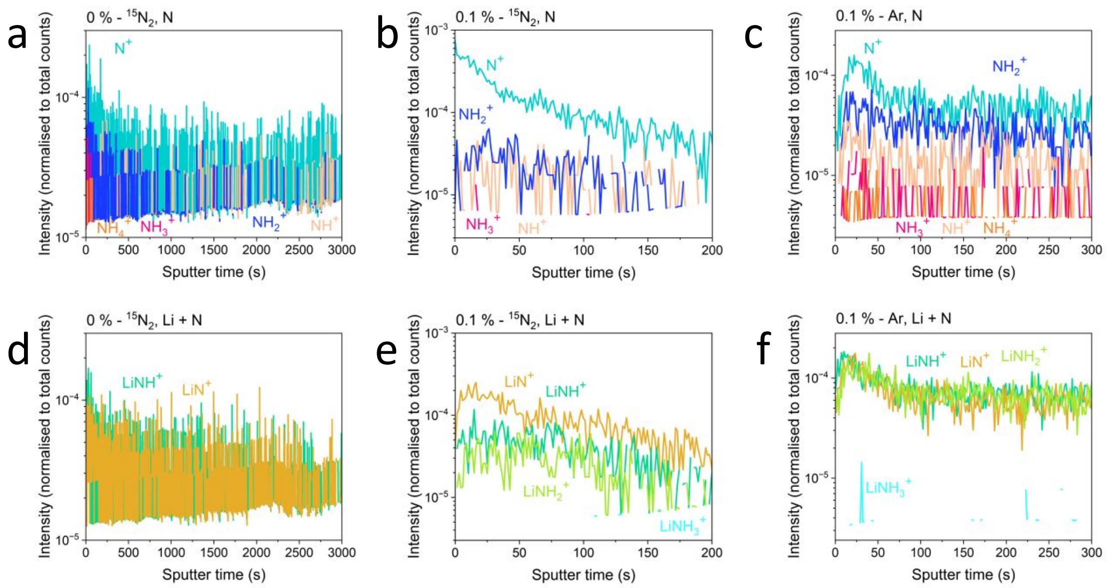

Figure S14 Time of Flight Secondary Ion Mass Spectrometry traces of Solid Electrolyte Interphase (SEI) samples formed in a 1 M $LiNTf_2$ in THF electrolyte with either 0 or 17 mM (0 or 0.1 vol %) ethanol added after passing -10 C at -2 mA cm^{-2} on a Mo electrode under 1 bar either $^{15}N_2$ or Ar. (a-c) N containing positive fragments for the 0 mM ethanol $^{15}N_2$, 17 mM (0.1 vol %) ethanol $^{15}N_2$, and 17 mM (0.1 vol %) ethanol Ar SEI samples respectively, (d-f) Li and N containing positive fragments for the 0 mM ethanol $^{15}N_2$, 17 mM (0.1 vol %) ethanol $^{15}N_2$, and 17 mM (0.1 vol %) ethanol Ar SEI samples respectively.

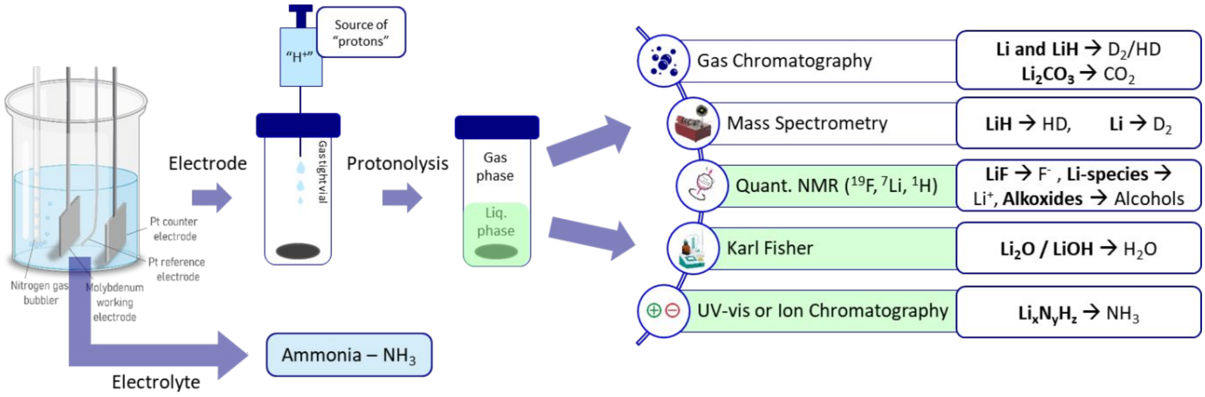

Figure S15 Workflow for the titration of electrode interphase species: a material of interest is placed in a sealed vial, to which is added a "titrant, a protic species (methanol-OD in the case of this work) that protonolyses the material's components, to yield analytes in the gas phase or liquid phase that can be quantified.

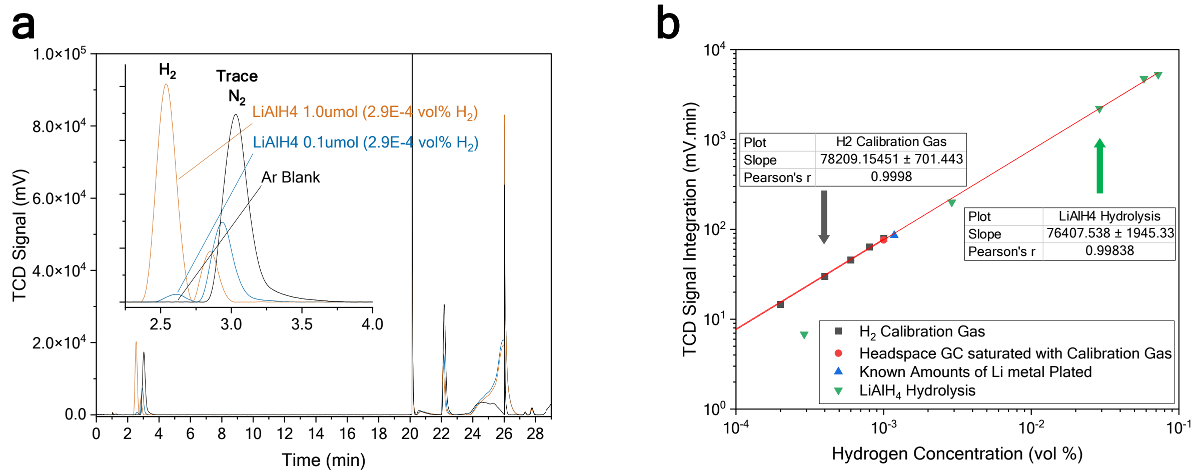

Figure S16. Calibration curves for GC quantification of hydrogen gas generated from Li and LiH protonolysis. (a) Typical GC chromatograms for hydrogen detection from a titrated sample: showing different amounts of $LiAlH_4$ hydrolysed with water alongside an Argon blank, measurements performed by Aishah Faisal and Dr Anna Winiwarter. Insert showing hydrogen peak. (b) Calibration curve for the quantification of hydrogen by different methods. In-line hydrogen gas flow calibration (black squares) and titration of known amounts of 1 M $LiAlH_4$ in THF with water (green triangles, performed by Aishah Faisal and Dr Anna Winiwarter). Calibrations proofed against headspace analysis of a known mixture of hydrogen gas in CO_2 (red circle), and titration of known amounts of lithium plated in a coin cell (blue triangle).

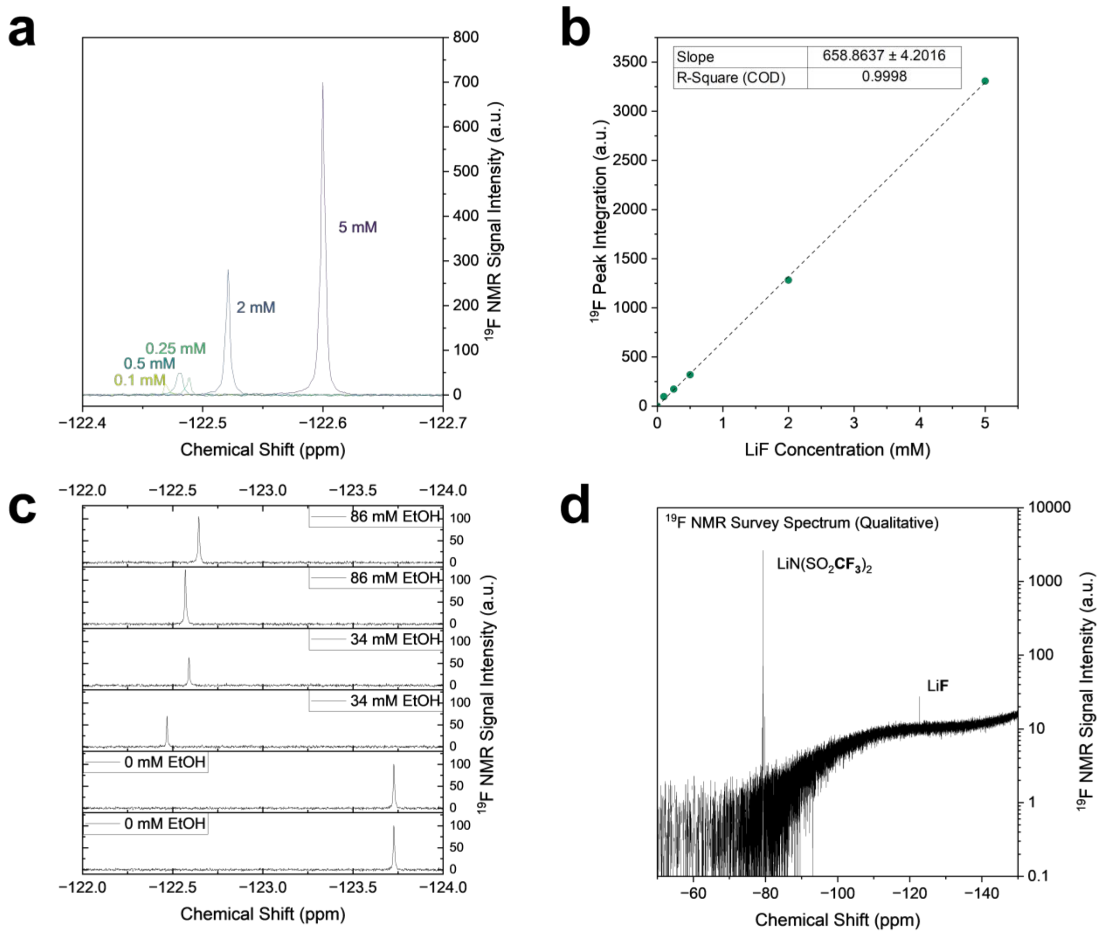

Figure S17. Chemical titration of LiF in the electrode deposits by (semi-)quantitative ^{19}F NMR. (**a**) Quantitative ^{19}F NMR spectra of different solutions of LiF in D_2O. (**b**) Calibration curve for the ^{19}F NMR integration of LiF dissolved in D_2O. Using the signal at ~-122.5 ppm corresponding to free F-ions, made by dissolving known amounts of LiF in D_2O and subjecting to the quantitative ^{19}F NMR titration method. (**c**) Quantitative NMR spectra for samples obtained from the dissolution in 1 ml D_2O of electrodes prepared during N_2 reduction experiments in the presence of different amounts of EtOH in the electrolyte. These spectra show some variation in the chemical shift for detected fluoride. However, a change of < 1.5 ppm is too small to represent significant matrix effects compromising the quantitative nature of the measurements[20]. (**d**) ^{19}F NMR qualitative survey spectra, detecting a peak at ~-80 ppm attributed to $-CF_3$ groups of unwashed $LiNTf_2$ salt, and LiF at ~-122 ppm. No other fluorinated product. NMR experiment developed with the help of Dr Stuart Elliott, NMR facility manager.

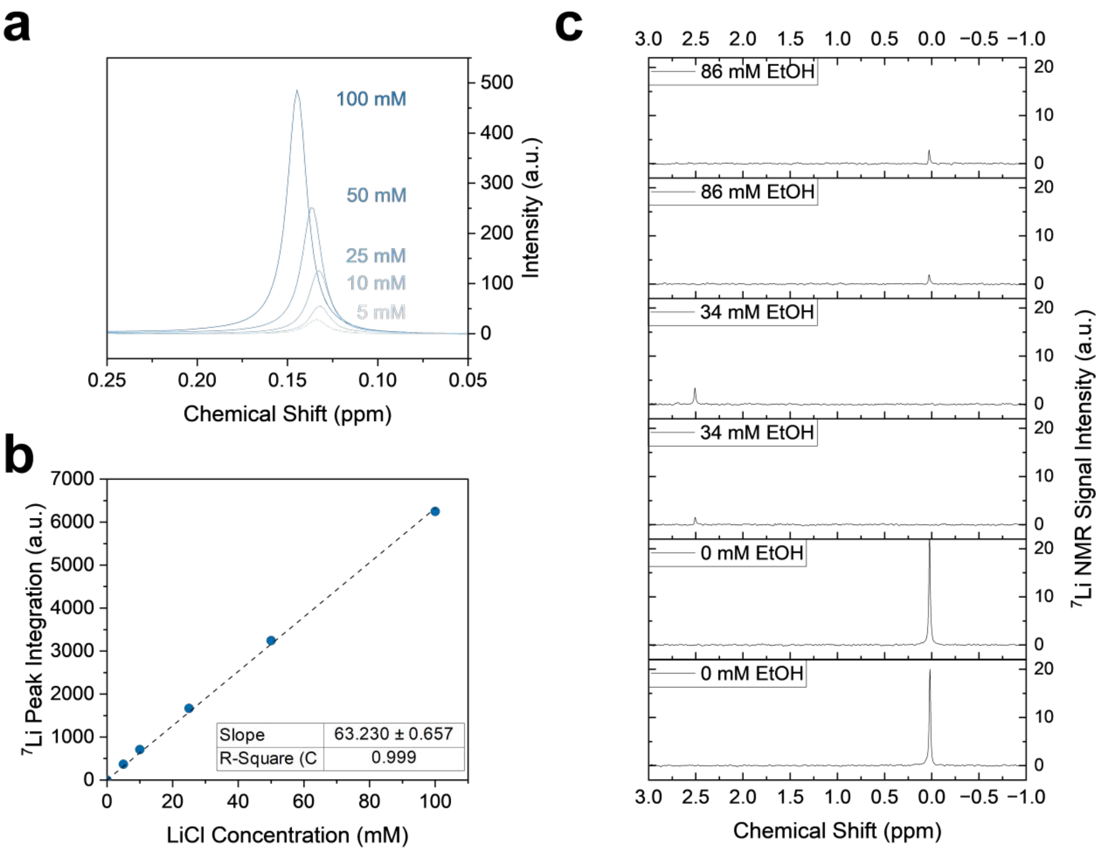

Figure S18 Chemical titration of Li^+ species in the electrode deposits by (semi-)quantitative ^{7}Li NMR. (**a**) Quantitative ^{7}Li NMR spectra of different solutions of LiF in D_2O. (**b**) Calibration curve for the ^{7}Li NMR integration of LiCl dissolved in MeOD:D_2O 1:9 v/v. Using the signal at ~0.15 ppm corresponding to dissolved Li-ions, made by dissolving known amounts of LiCl in MeOD:D_2O 1:9 v/v and subjecting to the quantitative ^{7}Li NMR titration method. (**c**) Quantitative NMR spectra for samples obtained from the dissolution in 1 ml MeOD of electrodes followed with dilution 1/10 in D_2O, prepared during N_2 reduction experiments in the presence of different amounts of EtOH in the electrolyte. These spectra show some variation in the chemical shift for detected Li^+, suggesting that the data might only be semi-quantitative. The relaxation times for the measurements were adjusted to make sure the signal remained quantitative despite this change. NMR experiment developed with the help of Dr Stuart Elliott, NMR facility manager.

References

(1) Andersen, S. Z.; Čolić, V.; Yang, S.; Schwalbe, J. A.; Nielander, A. C.; McEnaney, J. M.; Enemark-Rasmussen, K.; Baker, J. G.; Singh, A. R.; Rohr, B. A.; Statt, M. J.; Blair, S. J.; Mezzavilla, S.; Kibsgaard, J.; Vesborg, P. C. K.; Cargnello, M.; Bent, S. F.; Jaramillo, T. F.; Stephens, I. E. L.; Nørskov, J. K.; Chorkendorff, I. A Rigorous Electrochemical Ammonia Synthesis Protocol with Quantitative Isotope Measurements. *Nature* **2019**, *570* (7762), 504–508. https://doi.org/10.1038/s41586-019-1260-x.

(2) Nielander, A. C.; Blair, S. J.; McEnaney, J. M.; Schwalbe, J. A.; Adams, T.; Taheri, S.; Wang, L.; Yang, S.; Cargnello, M.; Jaramillo, T. F. Readily Constructed Glass Piston Pump for Gas Recirculation. *ACS Omega* **2020**, *5* (27), 16455–16459. https://doi.org/10.1021/acsomega.0c00742.

(3) Tort, R.; Bagger, A.; Westhead, O.; Kondo, Y.; Khobnya, A.; Winiwarter, A.; Davies, B. J. V.; Walsh, A.; Katayama, Y.; Yamada, Y.; Ryan, M. P.; Titirici, M.-M.; Stephens, I. E. L. Searching for the Rules of Electrochemical Nitrogen Fixation. *ACS Catal* **2023**, *13* (22), 14513–14522. https://doi.org/10.1021/acscatal.3c03951.

(4) Westhead, O.; Spry, M.; Bagger, A.; Shen, Z.; Yadegari, H.; Favero, S.; Tort, R.; Titirici, M.; Ryan, M. P.; Jervis, R.; Katayama, Y.; Aguadero, A.; Regoutz, A.; Grimaud, A.; Stephens, I. E. L. The Role of Ion Solvation in Lithium Mediated Nitrogen Reduction. *J Mater Chem A Mater* **2023**, *11* (24), 12746–12758. https://doi.org/10.1039/D2TA07686A.

(5) Oyakhire, S. T.; Gong, H.; Cui, Y.; Bao, Z.; Bent, S. F. An X-Ray Photoelectron Spectroscopy Primer for Solid Electrolyte Interphase Characterization in Lithium Metal Anodes. *ACS Energy Lett* **2022**, *7* (8), 2540–2546. https://doi.org/10.1021/acsenergylett.2c01227.

(6) Yu, W.; Lin, K.-Y.; Boyle, D. T.; Tang, M. T.; Cui, Y.; Chen, Y.; Yu, Z.; Xu, R.; Lin, Y.; Feng, G.; Huang, Z.; Michalek, L.; Li, W.; Harris, S. J.; Jiang, J.-C.; Abild-Pedersen, F.; Qin, J.; Cui, Y.; Bao, Z. Electrochemical Formation of Bis(Fluorosulfonyl)Imide-Derived Solid-Electrolyte Interphase at Li-Metal Potential. *Nat Chem* **2025**, *17* (2), 246–255. https://doi.org/10.1038/s41557-024-01689-5.

(7) Li, S.; Zhou, Y.; Li, K.; Saccoccio, M.; Sažinas, R.; Andersen, S. Z.; Pedersen, J. B.; Fu, X.; Shadravan, V.; Chakraborty, D.; Kibsgaard, J.; Vesborg, P. C. K.; Nørskov, J. K.; Chorkendorff, I. Electrosynthesis of Ammonia with High Selectivity and High Rates via Engineering of the Solid-Electrolyte Interphase. *Joule* **2022**, *6* (9), 2083–2101. https://doi.org/10.1016/j.joule.2022.07.009.

(8) Du, H.-L.; Chatti, M.; Hodgetts, R. Y.; Cherepanov, P. V.; Nguyen, C. K.; Matuszek, K.; MacFarlane, D. R.; Simonov, A. N. Electroreduction of Nitrogen with Almost 100% Current-to-Ammonia Efficiency. *Nature* **2022**, *609* (7928), 722–727. https://doi.org/10.1038/s41586-022-05108-y.

(9) Steinberg, K.; Yuan, X.; Klein, C. K.; Lazouski, N.; Mecklenburg, M.; Manthiram, K.; Li, Y. Imaging of Nitrogen Fixation at Lithium Solid Electrolyte Interphases via Cryo-Electron Microscopy. *Nat Energy* **2022**, *8* (2), 138–148. https://doi.org/10.1038/s41560-022-01177-5.

(10) Koh, H.; Das, S.; Zhang, Y.; Detsi, E.; Stach, E. A. Electron Beam-Induced Artifacts in SEI Characterization: Evidence from Controlled-Dose Diffraction Studies. *ACS Energy Lett* **2025**, *10* (1), 534–540. https://doi.org/10.1021/acsenergylett.4c03337.

(11) Cheng, D.; Lu, B.; Raghavendran, G.; Zhang, M.; Meng, Y. S. Leveraging Cryogenic Electron Microscopy for Advancing Battery Design. *Matter* **2022**, *5* (1), 26–42. https://doi.org/10.1016/j.matt.2021.11.019.

(12) Liang, K.; Yuan, X.; Chen, X.; Liu, B.; Kim, J. T.; Yu, J.; Zhao, D.; Li, Y. A Beginner's Guide to Cryo-EM for Battery Research. *Nano Lett* **2025**, *25* (18), 7210–7223. https://doi.org/10.1021/acs.nanolett.5c00740.

(13) Steinberg, K.; Gallant, B. M. Revealing the Role of Lithium Carbonate at Lithium Metal Anodes Through Study of Gas-Reacted Interphases. *J Electrochem Soc* **2024**, *171* (8), 080530. https://doi.org/10.1149/1945-7111/ad6d92.

(14) Fang, C.; Li, J.; Zhang, M.; Zhang, Y.; Yang, F.; Lee, J. Z.; Lee, M. H.; Alvarado, J.; Schroeder, M. A.; Yang, Y.; Lu, B.; Williams, N.; Ceja, M.; Yang, L.; Cai, M.; Gu, J.; Xu, K.; Wang, X.; Meng, Y. S. Quantifying Inactive Lithium in Lithium Metal Batteries. *Nature* **2019**, *572* (7770), 511–515. https://doi.org/10.1038/s41586-019-1481-z.

(15) Scott, S. B. Isotope-Labeling Studies in Electrocatalysis for Renewable Energy Conversion, and the Net Carbon Impact of This PhD Project. **2019**, No. July.

(16) Fearn, S. *An Introduction to Time-of-Flight Secondary Ion Mass Spectrometry (ToF-SIMS) and Its Application to Materials Science*; Morgan & Claypool Publishers, 2015. https://doi.org/10.1088/978-1-6817-4088-1ffirs.

(17) Peled, E.; Menkin, S. Review—SEI: Past, Present and Future. *J Electrochem Soc* **2017**, *164* (7), A1703–A1719. https://doi.org/10.1149/2.1441707jes.

(18) Bjarke Valbæk Mygind, J.; Pedersen, J. B.; Li, K.; Deissler, N. H.; Saccoccio, M.; Fu, X.; Li, S.; Sažinas, R.; Andersen, S. Z.; Enemark-Rasmussen, K.; Vesborg, P. C. K.; Doganli-Kibsgaard, J.; Chorkendorff, I. Is Ethanol Essential for the Lithium-Mediated Nitrogen Reduction Reaction? *ChemSusChem* **2023**. https://doi.org/10.1002/cssc.202301011.

(19) Du, H.-L.; Matuszek, K.; Hodgetts, R.; Dinh, K.; Cherepanov, P.; Bakker, J. M.; MacFarlane, D.; Simonov, A. N. The Chemistry of Proton Carriers in High-Performance Lithium Mediated Ammonia Electrosynthesis. *Energy Environ Sci* **2023**. https://doi.org/10.1039/D2EE03901J.

(20) Hobold, G. M.; Gallant, B. M. Quantifying Capacity Loss Mechanisms of Li Metal Anodes beyond Inactive Li 0. *ACS Energy Lett* **2022**, *7* (10), 3458–3466. https://doi.org/10.1021/acsenergylett.2c01845.
